# An Overview of the Mechanisms of HPV-Induced Cervical Cancer: The Role of Kinase Targets in Pathogenesis and Drug Resistance

**DOI:** 10.3390/cancers18020318

**Published:** 2026-01-20

**Authors:** Medha Karnik, SubbaRao V. Tulimilli, Preethi G. Anantharaju, Anjali Devi S. Bettadapura, Suma M. Natraj, Habeeb S. Mohideen, Sinisa Dovat, Arati Sharma, SubbaRao V. Madhunapantula

**Affiliations:** 1 Center of Excellence in Molecular Biology and Regenerative Medicine (CEMR) Laboratory (A DST-FIST Supported Center and ICMR-Collaborating Center of Excellence), Department of Biochemistry (A DST-FIST Supported Department), JSS Medical College, JSS Academy of Higher Education & Research (JSS AHER), Mysore 570015, Karnataka, India; medhakarnik07@gmail.com (M.K.); subbaraotulimilli23@gmail.com (S.V.T.); preethiganantharaju@jssuni.edu.in (P.G.A.); anjali.shrao@gmail.com (A.D.S.B.); suren156@gmail.com (S.M.N.); 2 Bioinformatics and Integrative Omics Lab, Department of Genetic Engineering, College of Engineering and Technology, SRM Institute of Science and Technology, Kattankulathur, Chengalpattu, Chennai 603203, Tamil Nadu, India; habeebm@srmist.edu.in; 3 Department of Pediatrics, Division of Hematology and Oncology, Penn State Cancer Institute, Hershey, PA 17033, USA; sxd30@psu.edu; 4 Department of Molecular and Precision Medicine, Center for Cannabis and Natural Product Pharmaceuticals (CCNPP), Penn State Cancer Institute, Hershey, PA 17033, USA; 5 Special Interest Group in Cancer Biology and Cancer Stem Cells (SIG-CBCSC), JSS Medical College, JSS Academy of Higher Education & Research (JSS AHER), Mysore 570015, Karnataka, India

**Keywords:** cervical cancer, HPV, kinases, chemoresistance

## Abstract

Despite a thorough understanding of the structure of human papillomavirus (HPV) and its genotypic variations (high-risk and low-risk variants), the mechanisms underlying HPV-induced cervical cancer (CC) pathogenesis and the molecular signatures of drug resistance remain to be fully understood. Accumulating evidence has shown the involvement of kinase targets in the induction of drug resistance in high-risk (HR) HPV-CC. Molecularly, the genome of high-risk HPV is reported to control the expression of host kinases. In particular, Aurora kinases A, B, and C (ARKA, ARKB, and ARKC), phosphotidylinositol–trisphosphate kinase (PI3K)-Akt, and Glycogen synthase kinase3-α/β (GSK3 α/β) promote the transformation of infected cells, and also enhance the resistance of cells to various chemotherapeutic agents such as nelfinavir and cisplatin. However, the precise mechanisms through which HPV activates these kinases are yet to be fully elucidated. Furthermore, there is still ambiguity surrounding whether targeting HPV-induced kinases along with HPV-targeted therapies (such as phytopharmaceuticals and PROTAC/CRISPR-CAS-based systems) synergistically inhibit cervical tumor growth. Given the critical role of kinases in the pathogenesis and treatment of CC, a comprehensive review of current evidence is warranted. This review aims to provide key insights into the mechanisms of HPV-induced CC development, the involvement of kinases in drug resistance induction, and the rationale for combination therapies to improve clinical outcomes.

## Simple Summary

Although the structure and variants of human papillomavirus (HPV) are well characterized, the mechanisms by which HPV drives cervical cancer (CC) progression and drug resistance remain poorly explored. Existing evidence suggests that high-risk HPV regulates host kinases such as Aurora kinases (A, B, C), PI3K-Akt, and GSK3α/β, which contribute to cancer cell transformation and resistance to drugs like nelfinavir and cisplatin. However, how HPV activates these kinases is still not fully understood. It also remains uncertain whether targeting these HPV-induced kinases, in combination with HPV-directed therapies such as phytopharmaceuticals or CRISPR-based systems, could improve treatment outcomes. This review explores the molecular basis of HPV-induced cervical carcinogenesis, the role of kinases in drug resistance, and the therapeutic potential of combined targeting strategies.

## 1. Introduction

Cervical cancer (CC) remains the fourth most commonly diagnosed malignancy in women globally, with approximately 660,000 new cases recorded in the year 2022 [1]. Despite being preventable through organized screening and vaccination programs, it continues to pose a significant public health challenge, particularly in low- and middle-income countries (LMICs) [2]. The World Health Organization (WHO) has prioritized the elimination of CC by defining the target of “achieving an incidence rate of fewer than four cases per 100,000 women annually” [3]. While high-income countries (HICs) have seen significant declines in CC incidence and mortality due to robust public health infrastructure and national-level HPV vaccination campaigns, LMICs continue to face systemic barriers such as limited access to HPV vaccines, lack of organized screening programs, inadequate healthcare infrastructure, and cultural stigma [4].

Australia and Canada report age-standardized incidence rates of around 7.1 per 100,000 women, and the United States has an adjusted rate of 11.5 per 100,000 women aged 15 to 75 [5,6]. These reductions are largely attributed to early adoption of the HPV vaccine and effective cytology or HPV-based screening protocols. Based on current projections, countries such as the United Kingdom and Norway are on track to achieve elimination by 2040 and 2039, respectively [7,8]. The age-standardized incidence and mortality rates in regions such as Africa, Melanesia, South America, Southeast Asia, and South-Central Asia range from 15.3 to 40.1 and 7.8 to 28.6 per 100,000 women, respectively [9]. Africa remains the epicenter of the global CC crises, where the disease represents the leading cause of cancer-related death among women, which is further aggravated by the coexisting HIV epidemic in this area. In sub-Saharan Africa and parts of India, HIV co-infection significantly increases the risk of HPV acquisition and persistence, and of cervical intraepithelial neoplasia (CIN), underscoring the need for integrated HIV-HPV screening and care programs [10]. Additional risk modifiers, such as early marriage, high parity, long-term oral contraceptive usage, tobacco exposure, and nutritional deficiencies, are prevalent in LMIC settings [11,12,13,14]. In this review, we discussed the results of a large proportion of studies based on in vitro cell culture models, genetically engineered cells, or cells ectopically expressing individual HPV oncogenes rather than infection with the complete virus. In many cases, viral gene expression levels, exposure duration, and experimental conditions may not fully recapitulate the physiological timing and magnitude of natural HPV infection. Consequently, not all findings summarized here are necessarily reflective of events occurring during bona fide HPV infection in vivo. The reported observations were derived from cell culture-based systems, animal models, or analyses of human cervical cancer tissues or biopsies, to aid interpretation of their biological and translational relevance.

### Co-Factors Contributing to Cervical Carcinogenesis

A variety of co-factors influence the persistence of HPV and its progression to high-grade cervical intraepithelial neoplastic lesions (HCILs) and invasive carcinoma [15]. One of the strongest modifying factors is immunosuppression, where women with compromised immune systems have a significantly increased risk of HPV persistence and CC, with studies indicating up to a fourfold higher incidence in this population compared to HIV-negative individuals [16]. Early-age sexual activity and a high number of lifetime sexual partners are often associated with increased exposure to HPV and reduced mucosal immunity, both of which elevate CC risk [17]. For instance, a study reported that initiating sexual activity before the age of 17 years or having multiple sexual partners markedly increases the likelihood of acquiring and sustaining HPV infection [18]. High parity has also been consistently linked to CC risk, with women who have had more than seven full-term pregnancies showing nearly a fivefold increased risk [19]. It is hypothesized that repeated cervical trauma and hormonal fluctuations during pregnancy are likely to facilitate viral integration into the host genome [20]. Hormonal influences also refer to long-term use of oral contraceptives. Studies have shown that women using oral contraceptives for more than ten years have a significantly elevated risk of developing CC. A large pooled analysis reported up to a fourfold increase in cervical cancer incidence in women who have used oral contraceptives for 10 years [21].

**Figure 1:**
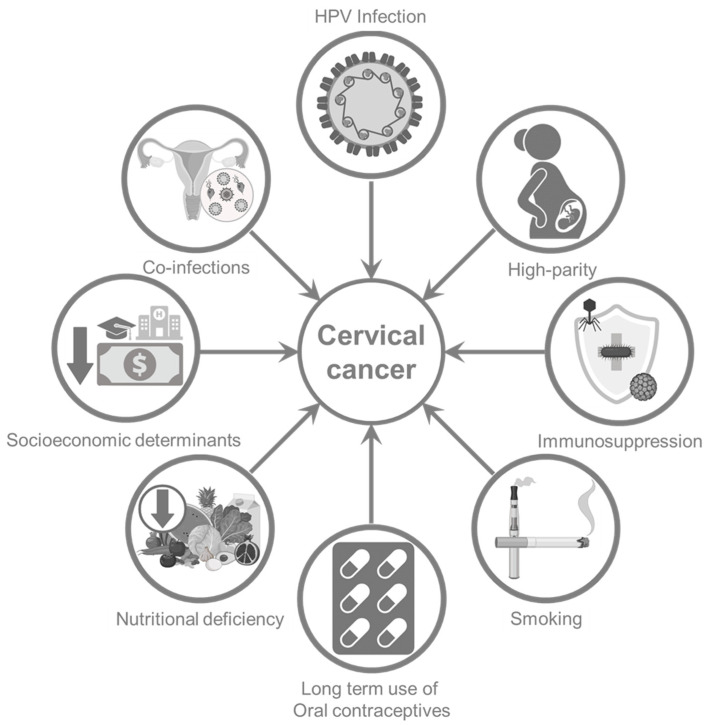
Major risk factors associated with cervical cancer. These include persistent infection with high-risk human papillomavirus, high parity, immunosuppression, tobacco smoking, long-term use of oral contraceptives, nutritional deficiency, socioeconomic determinants, and co-infections with pathogenic microorganisms.

Tobacco smoking represents another independent and well-established risk factor [22]. Carcinogenic compounds from tobacco smoke have been detected in cervical mucus, and smoking has been shown to impair local immune surveillance, especially by reducing Langerhans cell activity, thereby facilitating viral persistence and progression [23]. In addition, smokers with HPV infection are less likely to eliminate the virus compared to nonsmokers, further compounding their cancer risk. Co-infections with other sexually transmitted pathogens also play a significant role. Infection with Chlamydia trachomatis or Herpes simplex virus (HSV) type 2 has been associated with increased susceptibility to HPV persistence and progression to high-grade lesions [24]. Moreover, socioeconomic determinants such as low income, limited education, and reduced access to healthcare further increase CC risk by delaying HPV vaccination, screening, and treatment access [25]. Nutritional factors such as deficiencies in antioxidant vitamins A, C, and E are also likely to contribute to impaired epithelial repair and immune defense (Figure 1) [26,27,28].

Prophylactic vaccination against high-risk human papillomavirus (HR-HPV) genotypes, particularly HPV-16 and HPV-18, represents the most effective primary prevention strategy against CC [29]. These two genotypes are responsible for approximately 70% of CC cases worldwide [3]. The bivalent vaccine targeting HPV-16 and -18, the quadrivalent vaccine targeting HPV-6 and 11 [30], and the nonavalent vaccine targeting the nine HPV types [31] cover approximately 90% of oncogenic strains. The nonavalent vaccine, in particular, expands coverage to HPV-31, -33, -45, -52, and -58 types that are regionally significant in East Asia, Sub-Saharan Africa, and Latin America. Despite their proven efficacy, global vaccine coverage remains highly unequal (Table 1). As of 2022, more than 130 countries had introduced HPV vaccines into national immunization schedules; however, coverage in LMICs is substantially lower compared to that in HICs [32].

In summary, multiple biological and behavioral factors—including compromised immunity, early sexual initiation, high parity, prolonged contraceptive use, smoking, poor nutrition, and co-infections—contribute to the persistence and oncogenic progression of high-risk HPV infections. Timely vaccination against oncogenic HPV strains is therefore critical for reducing disease burden; however, vaccine coverage remains uneven globally. Expanded use of multivalent vaccines and comprehensive prevention strategies is essential to improve cervical cancer control.

## 2. Structural Features of HPV and Its Genome Organization

**Figure 2:**
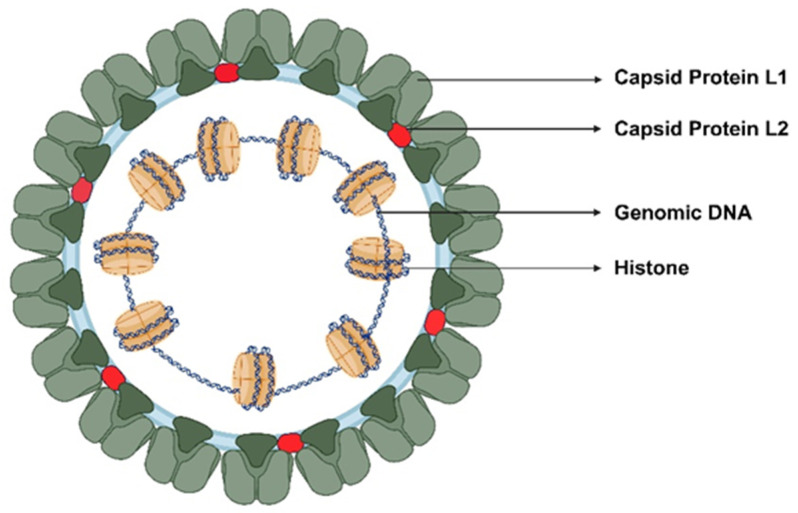
Structural components of human papillomavirus (HPV). HPV is a non-enveloped, icosahedral virus composed of a circular double-stranded DNA genome enclosed within a capsid formed of two structural proteins: L1 (major capsid protein) and L2 (minor capsid protein).

Human papillomavirus (HPV) is a small, non-enveloped virus with an icosahedral capsid measuring approximately 52–55 nm in diameter. It belongs to the Papillomaviridae family, which is a diverse group of epitheliotropic DNA viruses that infect the mucosal and cutaneous epithelium of vertebrates [36]. The HPV virion consists of 72 pentameric capsomers arranged in a T = 7 icosahedral capsid composed of major (L1) and minor (L2) structural proteins (Figure 1). L1 accounts for over 80% of the capsid and self-assembles into virus-like particles that form the basis of licensed HPV vaccines, while L2 facilitates viral genome packaging and endosomal escape during early infection (Figure 2) [37].

The viral genome is a circular, double-stranded DNA molecule spanning approximately 7900 to 8000 base pairs. It is tightly packed with histone-like cellular proteins, forming a nucleoprotein complex that mimics host chromatin. The genome is functionally organized into three regions: the early (E) region, the late (L) region, and the long control region (LCR), also referred to as the upstream regulatory region (URR) [38]. Each genomic region regulates distinct stages of the viral life cycle, including replication, transcription, and virion assembly. The early region encodes six proteins (E1–E7) expressed mainly in the basal and parabasal epithelial layers. Among these, E1 acts as a DNA helicase initiating viral replication, while E2 regulates early gene expression and facilitates recruitment of host replication machinery [39]. E4, although transcribed early, is expressed in the later stages of the viral life cycle and is implicated in viral genome amplification and virion release by disrupting cytoskeletal structures [40]. E5 contributes to immune evasion and cellular transformation by modulating growth factor receptor signaling and downregulating major histocompatibility complex (MHC) expression [41].

The early proteins E6 and E7 are central to HPV-mediated oncogenesis: E6 promotes ubiquitin-dependent degradation of p53, impairing apoptosis and DNA repair, while E7 inactivates pRb, releasing E2F transcription factors and driving unscheduled cell-cycle progression [42]. Together, E6 and E7 drive cellular immortalization and genomic instability, increasing susceptibility to malignant transformation. The late region encodes the structural proteins L1 and L2, expressed in differentiated epithelial layers during virion assembly, with L1 forming the outer capsid and mediating host cell attachment [43]. L2 supports viral genome encapsulation and facilitates trafficking through the endosomal and nuclear compartments of the host cell. Both L1 and L2 proteins contain conserved sequences that are targets for neutralizing antibodies, making them central components of prophylactic vaccine design [44].

The long control region (LCR) is a non-coding segment of approximately 800 to 900 base pairs situated between the L1 and E6 genes. It contains the origin of replication (ori) and multiple transcription factor binding sites, including those for AP-1, NF-1, and SP1, which regulate the expression of viral genes in a differentiation-dependent manner [45]. The LCR also harbors E2 binding sites that mediate transcriptional repression or activation depending on E2 protein concentration [46]. Through these regulatory elements, the LCR integrates host cellular signals and environmental cues to control the temporal expression of early- and late-stage genes during the viral life cycle. This region plays a pivotal role in coordinating replication with epithelial differentiation, ensuring the virus completes its life cycle as infected basal cells migrate and differentiate toward the epithelial surface [45]. The structural architecture and genomic organization of HPV are crucial for its successful infection and replication within the epithelial tissues. Understanding these molecular features will provide a foundation for targeted strategies in HPV prevention and therapeutic development.

## 3. HPV-Mediated Molecular Mechanisms in Cervical Carcinogenesis

Persistent infection with HR-HPV is recognized as the principal etiological factor in the development of CC [46]. Of the more than 200 known HPV genotypes, approximately 40 are capable of infecting the genital tract, and some of these are classified as carcinogenic, including types 16, 18, 31, 33, 35, 39, 45, 51, 52, 56, 58, 59, and 68 [47]. These HR-HPV types are grouped within specific phylogenetic clusters of the alpha genus, with species such as alpha-9, alpha-7, alpha-5, and alpha-6 harboring the most oncogenic genotypes [29]. It is estimated that over 90% of CC cases result from HPV infection, with high-risk types accounting for approximately 85% of them [48].

In the majority of women, HPV infections are temporary and are cleared by the immune system within one to two years [49]. However, around 10% of infections persist, and this persistence significantly reduces the likelihood of spontaneous viral clearance. Of those with persistent infections, approximately 1% to 4% may progress to cervical intraepithelial neoplasia (CIN) or CC over five or more years [50]. The risk of progression is strongly influenced by the HPV genotype and the individual’s immune status [51]. Immunocompromised women, including those with HIV infection or those who have undergone organ transplantation, are also particularly vulnerable [52].

One of the important events in the malignant transformation of cervical epithelial cells is the integration of HPV DNA into the host genome [53]. In the initial stages of infection, HPV DNA exists in an episomal state, and as persistence continues, the viral genome becomes embedded within the host’s chromosomes [54]. The integration of HPV DNA in the genome predominantly affects chromosomes 3q28, 8q24.21, 13q22.1, 17q23.1, and 1p36.32, with chromosomes 3, 8, and 1 accounting for the majority of events. These loci often contain key cancer-related genes, such as MYC on 8q24.21, and TP63 on 3q28, highlighting a functional link between integration sites and oncogenic transformation [55]. The frequency of HPV integration rises from 50% in precancerous lesions to more than 90% in invasive cervical carcinomas, with variability observed across HPV genotypes and histological subtypes [38]. Earlier categorizations recognized two major integration forms: one involving the insertion of a complete single viral genome, and the other characterized by tandem copies of the virus [56]. With the use of long-read sequencing platforms, additional patterns of integration have been uncovered that include rearranged or truncated viral genomes lacking E6 or E7 regions [57]. Both silent and transcriptionally active forms of integrated HPV DNA have been observed, each contributing in distinct ways to disease progression. 

Mechanistically, HPV integration frequently disrupts the E2 open reading frame, a regulator that normally constrains the expression of viral oncogenes [58]. Loss of E2 function leads to deregulated expression of E6 and E7 proteins, which impair key tumor suppressors p53 and the retinoblastoma (Rb) family proteins, respectively, facilitating evasion of cell-cycle checkpoints and apoptotic signaling [59]. Breakpoints are often located within short homologous sequences shared between the viral and host genomes, which are enriched in coding or regulatory regions, including microRNA loci. A substantial proportion of integration events occur within or near host genes, affecting approximately half of functional coding elements. Tumor suppressors, such as RAD51B, involved in double-stranded-DNA repair, have been shown to undergo amplification that spans both viral and host sequences, resulting in non-functional transcripts. Other genes, such as ETS2, FHIT, and LRP1B, have similarly been implicated as recurrent integration targets [60]. Conversely, HPV integration adjacent to oncogenes can lead to their transcriptional upregulation through mechanisms like local gene amplification. The insertion of viral sequences upstream of NR4A2 has been associated with extensive amplification and overexpression of this oncogene. Other loci, including MYC, HMGA2, ERBB2, and STARD3, are frequently altered in cervical tumors, further underscoring the oncogenic potential of integration-driven dysregulation [61]. The integration process occurs at fragile sites within the genome that are susceptible to DNA breaks and structural instability. These regions are hotspots for chromosomal alterations, including deletions, amplifications, and translocations [62]. In some cases, clonal integration events lead to inter-chromosomal rearrangements or the formation of extrachromosomal hybrid DNA elements composed of both viral and human sequences. These changes contribute to tumor heterogeneity and can accelerate malignant transformation [63]. HPV integration may also facilitate immune escape mechanisms. By altering viral gene expression, integrated genomes can downregulate the production of viral antigens, reducing their accessibility to the immune system. Additionally, E6 and E7 proteins interfere with host innate immune responses, including the suppression of antiviral signaling pathways. Interactions between viral elements and host transcriptional regulators such as specificity protein 1 (Sp1) have been shown to modify chromatin accessibility and antigen presentation [64]. These effects can impair immune recognition and clearance of infected cells, enabling persistent infection and neoplasia.

**Figure 3:**
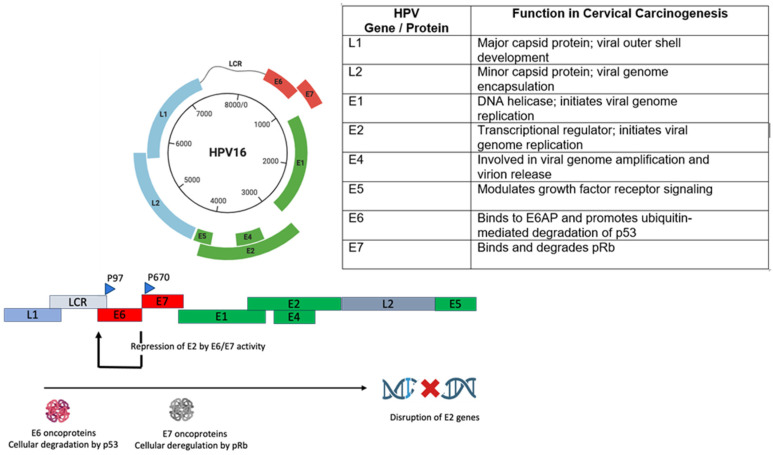
Oncoprotein-mediated molecular events in CC: This illustration represents the HPV16 genome organization and describes the oncogenic roles of E6 and E7, which interfere with tumor suppressor proteins (p53 and Rb), leading to cellular transformation and cancer development. L1 and L2 are structural proteins, while E1, E2, E4, E5, E6, and E7 are the regulatory and replication genes that play a role in viral lifecycle management.

Under physiological conditions, transcription of E6 and E7 is initiated at the P97 promoter, and their expression is modulated by the E2 protein, which binds to the URR and inhibits excessive transcription of these oncogenes [65]. The E2 gene itself is transcribed from the P670 promoter and represents an important regulatory checkpoint that prevents the deregulated expression of E6 and E7 [66]. However, during persistent infection and subsequent integration of the viral genome into the host cell’s DNA, a frequent event in high-grade lesions and CC, the E2 open reading frame is often disrupted [67]. This genomic integration typically occurs within or near the E2 region, leading to its functional inactivation. The loss of E2 function results in the uncontrolled expression of E6 and E7, tipping the balance toward oncogenesis [68]. The E6 oncoprotein exerts its tumorigenic effects primarily through the degradation of the tumor suppressor protein p53, a central regulator of the DNA damage response, apoptosis, and genomic integrity [69]. E6 facilitates the ubiquitin-mediated proteasomal degradation of p53, thereby impairing the cell’s ability to undergo apoptosis or arrest the cell cycle in response to DNA damage [70]. In parallel, the E7 oncoprotein targets the retinoblastoma protein (pRb), a key gatekeeper of the G1-to-S-phase transition. E7 binds to and promotes the degradation of pRb, leading to the release of E2F transcription factors, which drive the expression of genes necessary for S-phase entry and DNA synthesis. This disruption of the pRb-E2F axis results in uncontrolled cellular proliferation [71]. Collectively, the disruption of E2 due to viral integration and the subsequent overexpression of E6 and E7 represent a shift in the virus–host cell interaction, driving malignant transformation (Figure 3). The actions of E6 and E7 disable two major tumor suppressor pathways, p53 and pRb, leading to loss of genomic surveillance, increased cellular proliferation, and accumulation of genetic mutations [72]. Thus, the interplay between HPV integration, E2 inactivation, and oncogene overexpression constitutes a central mechanism in HPV-mediated carcinogenesis in CC. These HPV oncoprotein-mediated alterations are detectable during the dysplastic stages of cervical disease and precede the development of invasive cervical cancer, indicating that they represent early pathogenic events that may be amenable to pharmacological intervention before malignant transformation [73].

## 4. Kinase Pathways Targeted by E6 and E7 in Cervical Pathogenesis

High-risk HPV oncoproteins E6 and E7 actively influence host cell kinase signaling networks to support viral persistence and malignant transformation. By manipulating key pathways such as Aurora kinases A, B, and C (ARKA, ARKB, and ARKC), PI3K/Akt/mTOR, MAPK/ERK; Wnt/β-catenin; JNK/p38 MAPK; and PKC-mediated signaling, these viral proteins induce cell proliferation, inhibit apoptosis, promote metabolic reprogramming, and enable evasion of immune surveillance [74,75,76,77]. Many studies have elucidated the intricate mechanisms through which E6 and E7 use these kinase networks, offering new insights into potential therapeutic options.

### 4.1. Involvement of Aurora Kinase Pathway in the Pathogenesis of Cervical Cancer

Aurora kinase A (ARKA) plays a critical role in the development of CC, particularly through its interaction with the E6 oncoprotein [78]. Overexpression of ARKA contributes to chromosomal instability and promotes tumorigenesis by facilitating the degradation of the tumor suppressor protein p53 through phosphorylation-dependent ubiquitination, as demonstrated primarily in HPV-positive cervical cancer cell line models [79]. ARKA is reported to be elevated in ~80% of CC cases, and this overexpression is linked to more aggressive tumor behavior and unfavorable prognosis [80]. Guo et al. showed the physical interaction between the HPV E6 oncoprotein and ARKA. Silencing of E6 led to a reduction in both Aurora A mRNA and protein expression, and conversely, Aurora A knockdown also caused a decline in E6 levels. These results indicate a mutual stabilization mechanism, where the interaction between E6 and Aurora A helps in maintaining the expression and stability of both proteins [80]. Similarly, ARKB is overexpressed in approximately 60% of cancers associated with HPV, where it plays a significant role in promoting genomic instability [81]. ARKB is activated through phosphorylation at multiple sites by different regulatory proteins, including Checkpoint kinase 1 (Chk1), Inner Centromere Protein (INCENP), and Kinetochore Scaffold 1 (KNL1), which are essential for proper cell division [82,83,84]. Specifically, Chk1 phosphorylates Aurora B at serine 331 to trigger its activation [85], while INCENP promotes autophosphorylation at threonine 232 [86]. In HPV-positive cancers, phosphorylated Aurora B is elevated, even though total Aurora B protein levels remain unchanged, suggesting enhanced activation rather than increased expression [87]. Comprehensive analysis of CC mRNA profiles has revealed that ARKA plays a central role within the altered gene expression networks, acting as a key regulatory node based on transcriptomic analyses of cervical cancer cohorts [88]. This suggests that ARKA may help address the challenge of tumor heterogeneity in CC. While less studied than Aurora A and B, emerging evidence suggests that AURKC is upregulated in certain HPV-positive CC cells and may contribute to chromosomal instability and aneuploidy [89]. E6 and E7 may facilitate AURKC overexpression by disrupting cell-cycle regulation and promoting mitotic defects.

AURKA plays a critical role in HPV E6- and E7-mediated CC metastasis through multiple mechanisms. A human kinome screen identified that inhibiting AURKA with the selective inhibitor Alisertib triggered apoptosis in E7-expressing CC cell lines. This occurred through prolonged mitotic arrest, downregulation of the anti-apoptotic protein Mcl-1, and stabilization of the pro-apoptotic protein BIM. In mouse xenograft models, Alisertib treatment led to tumor regression, consistently preventing regrowth even after the treatment concluded [90]. Li et al. showed that E6 binds to AURKA through its C-terminal region, enhancing AURKA expression and activity. This interaction promotes cyclin E and phospho-Histone H3 expression, driving cell-cycle progression and supporting proliferation and invasion in HPV-positive CC cells. These findings suggest that the E6–AURKA complex contributes to oncogenesis and may represent a therapeutic target [91]. Together, these studies suggest that E6 and E7 help stabilize AURKA and increase the ability of HPV-positive CC cells to grow, spread, and become more aggressive.

**Figure 4:**
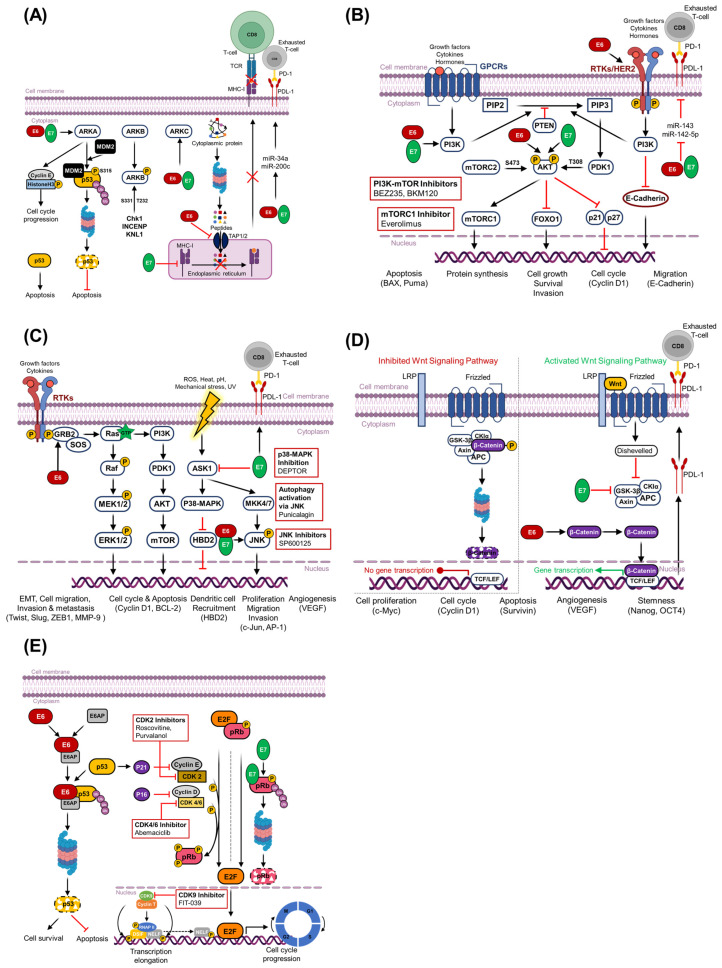
Molecular pathways altered by HPV in cancer progression and therapeutic targets. (**A**) Aurora kinase signaling: HPV oncoproteins E6 and E7 upregulate Aurora kinase A (AURKA) and Aurora kinase C (AURKC). Aurora kinase B (AURKB) is activated by regulatory proteins Checkpoint kinase 1 (Chk1), Inner Centromere Protein (INCENP), and Kinetochore Scaffold 1 (KNL1). E6 and E7 stabilize AURKA, promoting cervical carcinogenesis by phosphorylating and inducing ubiquitin-mediated degradation of p53. (**B**) PI3K/Akt/mTOR signaling: E6 and E7 drive persistent activation of the PI3K/Akt/mTOR pathway, contributing to cervical cancer development and progression. (**C**) MAPK/ERK signaling: E6 and E7 also mediate continuous activation of the MAPK/ERK pathway, facilitating tumorigenesis. (**D**) Wnt/β-Catenin Pathway: E6 and E7 modulate the Wnt/β-catenin pathway, enhancing transcription of oncogenic target genes and promoting cell proliferation, stemness, angiogenesis, and metastasis. (**E**) Cyclin-Dependent Kinases (CDKs): Disruption of pRb and associated cyclin-dependent kinase regulation by E7 removes cell-cycle brakes, while E6-mediated p53 degradation further deregulates the cell cycle.

E6 and E7 exert immune evasion strategies in CC, antagonizing both innate and adaptive immunity. E6 suppresses antigen processing by diminishing TAP1/2 expression, reducing presentation of viral peptides on MHC class I, and impairing CD8^+^ T-cell recognition [92]. E7 contributes to immune evasion in CC by downregulating MHC class I surface expression, thereby impairing antigen presentation to cytotoxic T lymphocytes and facilitating immune escape. However, this reduction in MHC-I may enhance tumor cell visibility to natural killer cells, which recognize and target cells with low MHC-I expression. Additionally, both E6 and E7 have been shown to upregulate PD-L1 expression through the modulation of specific microRNAs, such as miR-142–5p, establishing an immunosuppressive tumor microenvironment that promotes T-cell exhaustion and supports tumor progression [93]. A recent study demonstrated that combining alisertib with anti-CTLA-4 immunotherapy significantly enhances antitumor immunity in HPV-positive cancers. In an HPV-driven murine model, this combination therapy reduced tumor size and improved survival, with 75% of treated mice surviving beyond 60 days. Alisertib induced apoptosis, DNA damage, and immunogenic cell death while also enhancing T-cell activity and reducing immune suppression. RNA-seq revealed upregulation of immune-related genes and metabolic reprogramming, indicating that alisertib reshapes the tumor immune microenvironment to overcome immune resistance and improve therapeutic outcomes (Figure 4A) [94].

### 4.2. The PI3K/Akt/mTOR Pathway Drives the Survival of Cervical Cancer Cells and Promotes Resistance to a Spectrum of Pharmacological Agents

The PI3K/Akt/mTOR pathway serves as a pro-survival factor in HPV-positive cells, with both E6 and E7 engaging this axis via complementary mechanisms that support viral persistence, enhance proliferation, and facilitate resistance to cellular stress, driving cervical and other HPV-associated cancers [95]. Bossler et al. demonstrated that under hypoxia, HPV-positive cancer cells enter a reversible dormancy, which is characterized by strong repression of E6/E7 oncogenes. They showed that this downregulation is mediated by AKT activation via the PI3K/mTORC2 pathway, which operates in hypoxia, but not under normoxic conditions. Chemical inhibition of AKT, PI3K, or mTORC2 reverses E6/E7 repression, underscoring the centrality of this signaling axis in hypoxic tumor adaptation [96]. Lee et al. showed that HPV16 E6 upregulates the receptor tyrosine kinase Axl via the PTEN/AKT signaling axis in CC. E6 knockdown restored Membrane-Associated Guanylate Kinase 2 (MAGI-2), increased PTEN phosphorylation, and reduced AKT activation, confirming the regulatory role of EAxl silencing inhibited metastasis and improved NK cell-mediated cytotoxicity. Clinically, Axl expression correlated with HPV16/18 infection and advanced CC stages [97]. HPV-16 E7 was found to enhance phospholipase D (PLD) activity in host cells by promoting degradation of pRb, leading to increased production of phosphatidic acid, a key regulator of the PI3K/AKT/mTOR pathway. This modulation reduced cellular sensitivity to Rapamycin, an mTOR inhibitor, thereby conferring a growth advantage. Cells expressing E7 or with silenced pRb show resistance to Rapamycin’s antiproliferative effects. These findings indicate that targeting both PLD and mTOR pathways may offer a more effective therapeutic approach in HPV-driven malignancies [98]. In HPV E7-expressing cells such as SiHa, S11 phosphorylation is found to be driven not by CDK1, but by activation of the PI3K/AKT/mTOR pathway. This phosphorylation occurs throughout the cell-cycle, during mitosis, and is essential for bypassing the G1 checkpoint. Mutation of RCC1 at S11 to a non-phosphorylatable form impairs its ability to promote G1/S transition, highlighting its functional relevance. These findings reveal a novel mechanism by which HPV E7 manipulates host cell cycle progression via PI3K/AKT/mTOR-mediated phosphorylation of RCC1 [99]. Zhang et al. demonstrated that HPV11 E6 activates autophagy in keratinocyte-derived cells by inhibiting key autophagy-suppressing pathways. They showed that E6 expression leads to decreased phosphorylation of mTOR at Ser2448, along with reduced phosphorylation of AKT and Erk, indicating suppression of the AKT/mTOR and Erk/mTOR pathways. Knockdown of E6 reversed these effects, confirming its role in the progression of CC. These findings reveal a novel mechanism by which lr-HPV11 regulates autophagy, contributing to the understanding of LR-HPV pathogenesis [100]. All these studies collectively highlight the central role of the PI3K/Akt/mTOR pathway in mediating HPV-induced cellular transformation, survival, and therapy resistance in both HR- and LR-HPV-associated diseases. E6 and E7 oncoproteins activate this signaling axis through distinct but complementary mechanisms, enabling persistent viral expression, enhanced proliferation, and resistance to stress and immune responses, thus facilitating the development and progression of cervical and other HPV-related cancers.

In CC, the PI3K/Akt/mTOR signaling pathway is critically implicated in metastasis. E6/E7-mediated activation of PI3K/Akt leads to downregulation of E-cadherin and disruption of adherens junctions, affecting intercellular adhesion and promoting detachment of tumor cells. Hu et al. investigated the regulatory role of E6/E7 oncoproteins in driving EMT in CC through the PI3K/Akt pathway. The authors of this study found an inverse correlation between E-cadherin and E6/E7 expression, alongside a strong positive correlation with P-cadherin. Functional assays in CC cell lines revealed that modulation of E6/E7 expression significantly impacted cellular proliferation, migration, and invasion. Silencing E6/E7 via siRNAs restored E-cadherin levels and suppressed P-cadherin expression and was validated in in vivo models using a BALB/c-nu mouse xenograft model. These results suggest that HPV-16 E6/E7 promotes EMT and metastatic progression through AKT in CC through the induction of a cadherin switch [101]. E6/E7-driven mTOR activation enhances hypoxia-inducible factor 1-alpha (HIF-1α) expression even in normoxic conditions, which promotes angiogenesis and upregulates C-X-C chemokine receptor type 4 (CXCR4), a chemokine receptor involved in organ-specific metastasis, particularly to lymph nodes [102]. Inhibition of the PI3K/Akt and ERK1/2 signaling pathways was shown to suppress the accumulation of HIF-1α protein and the secretion of VEGF, which are otherwise induced by E6 and E7 oncoproteins in CC [102]. Collectively, all these studies highlight a complex network wherein E6 and E7 hijack the PI3K/AKT/mTOR pathway to enhance CC metastasis.

The HPV oncoproteins E6 and E7 also play a pivotal role in promoting immune evasion by hijacking the PI3K/AKT/mTOR signaling axis in CC. The E6 oncoprotein stabilizes HER2 via Hsp90, activating downstream PI3K/AKT signaling that leads to upregulation of PD-L1, thereby impairing T-cell-mediated cytotoxic responses. The E6 oncoprotein stabilizes HER2 via Hsp90, activating downstream PI3K/AKT signaling that leads to upregulation of programmed death-ligand 1 (PD-L1), thereby impairing T-cell-mediated cytotoxic responses [103]. Mutations in PIK3CA result in constitutive AKT/mTOR pathway signaling, which independently enhances PD-L1 expression and promotes the recruitment of immunosuppressive cells within the tumor microenvironment [104]. Additionally, E6 represses miR-143, leading to the accumulation of hypoxia-inducible factor-1α (HIF-1α), which transcriptionally upregulates PD-L1 expression even in normoxia [105]. In hypoxic tumor regions, activation of PI3K/mTORC2 and AKT contributes to the repression of E6/E7 expression, thereby reducing the levels of viral antigens presented to immune cells while maintaining cell survival, representing a novel adaptation for immune escape [106]. E6 and E7 were shown to downregulate miR-142-5p, which releases a block on PD-L1 and enables immune escape, which was confirmed in both in vitro and in vivo tumor models [107]. Collectively, these studies show how the E6/E7–PI3K/AKT/mTOR network drives immune evasion in CC, encompassing immune checkpoint upregulation and impaired T-cell function. However, more studies are required to identify therapeutic strategies combining AKT/mTOR pathway inhibitors with PD-1/PD-L1 blockade to enhance anti-tumor immunity in CC.

The PI3K/Akt/mTOR pathway is constitutively activated through a combination of HPV oncoprotein-mediated mechanisms and dysregulation of key upstream regulators [107]. This pathway is primarily initiated by extracellular stimuli, including growth factors (EGF, PDGF, IGF-1), cytokines, and hormones that bind to their cognate receptor tyrosine kinases (RTKs) such as epidermal growth factor receptor (EGFR), platelet-derived growth factor receptor (PDGFR), and insulin-like growth factor 1 receptor (IGF-1R) [108]. Ligand binding induces RTK dimerization and autophosphorylation at specific tyrosine residues within their cytoplasmic domains, creating docking sites for the Src homology 2 (SH2) domains of the p85 regulatory subunit of class IA PI3K [96]. This recruitment brings the p85-p110 heterodimer to the plasma membrane, where the p110 catalytic subunit phosphorylates phosphatidylinositol 4,5-bisphosphate (PIP2) to generate the second messenger phosphatidylinositol 3,4,5-trisphosphate (PIP3) [109].

In CC, multiple mechanisms converge to hyperactivate this pathway. Approximately 20–30% of CC cases harbor activating mutations in PIK3CA, the gene encoding p110α, with the most common hotspots being E542K, E545K (helical domain), and H1047R (kinase domain), rendering PI3K constitutively active independent of upstream signals [98]. Additional upstream elements include G protein-coupled receptors (GPCRs), which commonly activate p110β, and small GTPases like Ras, which in turn directly interact with and activate PI3K catalytic subunits [110]. Collectively, these upstream alterations converge on the generation of PIP3, which recruits AKT and PDK1 to the membrane through their PH domains, enabling PDK1 to phosphorylate AKT at Thr308, and mTORC2 to phosphorylate Ser473, resulting in full activation of AKT, which promotes cell growth, survival, and invasion, leading to CC progression [111].

Downstream effectors in the PI3K/Akt/mTOR pathway play a critical role in mediating the oncogenic functions leading to CC. Key downstream effectors include mTOR, which enhances protein synthesis and cell growth through activation of S6 kinase (S6K) and inhibition of 4EBP1, a translational repressor [112]. AKT also phosphorylates and inhibits BAD and caspase-9, thereby preventing apoptosis in CC [113]. E6 promotes p53 degradation via the E6AP ubiquitin ligase, triggering oncogenic cascades through key downstream effectors: (1) cell-cycle regulators (p21, cyclin D1) [103], (2) pro-apoptotic factors (Bax, Puma) [114], (3) DNA repair proteins (ATM, BRCA1) [105], and (4) metabolic sensors (AMPK, TIGAR) [115]. These effector disruptions collectively impair genomic stability, apoptosis, and growth suppression while activating PI3K/AKT. Forkhead box O (FOXO) transcription factors are critical downstream targets of the PI3K/AKT signaling pathway. Akt phosphorylates FOXO1/3a at three conserved residues (Thr24, Ser256, and Ser319 in FOXO1), inducing 14-3-3-mediated nuclear export and proteasomal degradation [116]. Metabolic reprogramming is facilitated through Akt’s direct phosphorylation of ATP-citrate lyase (ACLY) at Ser455, which increases acetyl-CoA production to support lipid synthesis and chromatin modifications essential for cancer cell proliferation [117]. Overall, the dysregulation of PI3K/AKT downstream effectors plays an important role in CC pathogenesis by promoting cell growth, inhibiting apoptosis, and enabling tumor progression, thereby highlighting this pathway as a critical target for therapeutic intervention in CC (Figure 4B).

### 4.3. Involvement of MAPK/ERK Pathway in the Malignant Transformation of Cervical Cancer Cells

The mitogen-activated protein kinase (MAPK)/ extracellular signal-regulated kinase (ERK) pathway is another signaling cascade regulated by high-risk HPV oncoproteins E6 and EStudies have shown that E6 induces phosphorylation of JNK1/2, which amplifies epidermal growth factor receptor (EGFR) signaling, subsequently activating the MAPK/ERK cascade [118]. This promotes key oncogenic processes, including cell proliferation, survival, and epithelial–mesenchymal transition (EMT), while sustaining E6/E7 expression, thereby establishing a feed-forward regulatory loop, which is essential for the progression of CC [119]. E6 oncoproteins from both hr- and lr-HPV types were found to enhance eukaryotic translation initiation factor 4E (eIF4E) phosphorylation at Ser209, primarily through the activation of the PI3K/AKT pathway and phosphorylation of eukaryotic translation initiation factor 4E binding protein 1 (4EBP1). Inhibition of eIF4E phosphorylation led to reduced expression of its oncogenic targets, Cyclin D1 (CCND1) and Ornithine Decarboxylase 1 (ODC1), suggesting a key role for E6 in modulating cap-dependent translation via AKT and ERK signaling. This highlighted a novel mechanism by which HPV E6 promotes oncogenesis through translational control of growth-related genes [120]. Bi et al. demonstrated that MAGT1, a magnesium transporter involved in ion homeostasis and glycosylation, is essential for cell proliferation in HPV-positive CC. In HeLa and SiHa cells, MAGT1 knockdown caused S-phase arrest and apoptosis, associated with dysregulation of key cell-cycle genes [121]. Transcriptomic profiling indicated MAGT1’s involvement in the MAPK signaling pathway, which was supported by reduced phosphorylation of ERK1/2 and p38 upon MAGT1 silencing [122]. Together, these findings highlight the diverse mechanisms by which HPV manipulates MAPK/ERK pathways, as well as translational control to support malignancy in CC. However, further studies are warranted to fully elucidate these pathways and assess their therapeutic potential.

E6 oncoprotein, especially the 83V variant (L83V), has been shown to increase ERK1/2 phosphorylation via the Rap1 pathway, promoting a more invasive cellular phenotype that enhances metastatic potential in cervical epithelial cells [123]. E6 and E7 activate the MEK/ERK cascade, which leads to the upregulation of EMT markers such as Slug; Twist; and ZEB1, VEGF, and IL-8, thereby facilitating tumor cell migration and invasion through both EMT and enhanced vascular support [124]. Estrogen signaling via ER-α36 enhances MAPK/ERK phosphorylation, further promoting migration and invasion in CC cell models [125]. Furthermore, E6 destabilizes the histone demethylase KDM5C, leading to super-enhancer activation of EGFR and c-MET, both of which signal through MAPK/ERK to drive proliferation and metastatic traits in CC cells [126]. The loss of PP2A phosphatase activity, a critical negative regulator of MAPK signaling, leads to sustained ERK phosphorylation in cervical cancer cells. This dysregulation enhances the expression of matrix metalloproteinase-9 (MMP-9), thereby promoting extracellular matrix degradation and contributing to a more invasive and metastatic phenotype in HeLa cells [127]. In CC, E6/E7 oncoproteins activate the MAPK/ERK signaling pathway, promoting key steps in metastasis. This activation enhances EMT and supports angiogenesis. Collectively, MAPK/ERK-driven changes facilitate tumor cell invasion, migration, and metastatic spread of CC.

E6 and E7 exploit the MAPK/ERK signaling axis to evade host immunity in CC through several key mechanisms. In CC, E7-driven activation of the ERK pathway leads to increased PD-L1 expression, enhancing immune checkpoint signaling and effectively dampening cytotoxic T-cell activity [128]. E7 suppresses the Apoptosis Signal-Regulating Kinase 1–p38 Mitogen-Activated Protein Kinase (ASK1–p38 MAPK) signaling pathway, leading to reduced expression of the antimicrobial peptide Human Beta-Defensin 2 (HBD2), which normally plays a key role in recruiting dendritic cells and initiating innate immune responses. As a result, early immune surveillance is weakened, allowing HPV-infected CC to evade detection [129]. E7 activates the ERK1/2, JNK, and p38 MAPK pathways in plasmacytoid dendritic cells, disrupting their normal function and preventing them from maturing properly. This leads to reduced production of type I interferons, which are critical for mounting an effective antiviral immune response [130]. E7 inhibits the MAPK phosphatase Dual Specificity Phosphatase 5 (DUSP5), removing the natural brake on ERK signaling in epithelial cells [131]. This leads to sustained ERK activation, which disrupts antiviral defense mechanisms and helps infected cells evade the innate immune response. E7 disrupts the host’s innate immune defenses by manipulating MAPK signaling in both immune and epithelial cells. In HeLa and CaSki cells, E7 promotes the upregulation of the histone methyltransferase SUV39H1, which epigenetically silences key antiviral sensors such as Retinoic acid-Inducible Gene I (RIG-I), Cyclic GMP-AMP Synthase (cGAS), and Stimulator of Interferon Genes (STING). This repression leads to reduced production of type I interferons, allowing HPV-infected cells to evade immune detection [132].

Upstream effectors of the MAPK/ERK signaling pathway play a crucial role in the initiation and progression of CC. E6 induces ligand binding, RTK dimerization, and autophosphorylation on specific tyrosine residues within the cytoplasmic domain of the receptors. These phosphotyrosine motifs serve as docking sites for adaptor proteins, particularly GRB2 (growth factor receptor-bound protein 2), which constitutively associates with the guanine nucleotide exchange factor SOS (son of sevenless) [133]. The GRB2–SOS complex then interacts with membrane-bound RAS, leading to the exchange of GDP for GTP and subsequent activation of upstream RAS. Activated RAS undergoes a conformational change and initiates the recruitment and activation of the serine/threonine kinase RAF, which phosphorylates and activates MAPK/ERK [134]. IL-1β has been shown to stimulate the MEK/ERK pathway in HeLa cells, increasing levels of phosphorylated MEK and ERK and promoting proliferation and migration [135]. Furthermore, TNF-α, frequently elevated in the tumor microenvironment, has been implicated in ERK activation through both NF-κB–dependent and MAPK-mediated mechanisms, contributing to chronic inflammation and tumor progression [136].

In the nucleus, ERK phosphorylates and activates transcription factors such as Cellular Finkel–Biskis–Jinkins Osteosarcoma (c-Fos), Cellular Jun Oncogene (c-Jun), ETS Like-1 (ELK1), and Myelocytomatosis oncogene (MYC), which regulate the genes cyclin D1, B-cell lymphoma 2 (BCL-2), and Myeloid Cell Leukemia 1(MCL-1) [137]. Moreover, ERK-mediated phosphorylation of TWIST and SNAIL contributes to EMT, which enhances invasion and metastasis [138]. ERK directly phosphorylates and activates downstream kinases such as p90RSK and MAPK-Interacting Kinase (MNK), which in turn regulate translation and protein synthesis, and also modulates focal adhesion kinase (FAK) to enhance cytoskeletal reorganization and cell motility [139]. ERK-mediated phosphorylation activates MNKs that phosphorylate the translation initiation factor eIF4E, thereby enhancing cap-dependent translation of growth-promoting mRNAs [140]. ERK signaling also enhances protein synthesis through the activation of MNK1/2 kinases, which are directly phosphorylated by ERK. Once activated, MNK1 and 2 phosphorylate the translation initiation factor eIF4E at Ser209, enhancing cap-dependent translation of mRNAs encoding proteins involved in cell proliferation, survival, and metastasis. This phosphorylation event has been consistently implicated in tumorigenesis, as MNK-mediated eIF4E activation promotes malignant phenotypes and resistance to stress across multiple cancer types [141]. Collectively, these downstream effectors of ERK play an important role in sustaining the malignant phenotype of CC cells by promoting cellular proliferation, enhancing protein synthesis, driving cytoskeletal remodeling, and facilitating tumor cell motility and invasion (Figure 4C).

### 4.4. Wnt/β-Catenin Pathway

The Wnt/β-catenin signaling pathway has emerged as a critical mediator of HPV-induced CC, and the HPV E6 and E7 oncoproteins manipulate this pathway. Lichtig et al. were the first to show that the E6 of HPV16 can activate β-catenin/TCF-dependent transcription through a mechanism that relies on its interaction with E6-associated protein (E6AP), suggesting a functional connection between HPV infection and β-catenin signaling in CC cells [142]. E6 promotes β-catenin stability and nuclear localization by disrupting its degradation via E6AP and reducing Siah-1/β-TrCP activity, while E7 interferes with PP2A to inhibit GSK3β, which further enhances β-catenin stabilization [76]. E6-induced transcription factors such as Myeloid Zinc Finger 1–NK2 Homeobox 1–Forkhead Box M1 (MZF1–NKX2-1–FOXM1) enhance β-catenin nuclear translocation and transactivation of target genes, including c-Myc and Cyclin D1, driving proliferation, invasion, and stemness in CC [143]. Calvillo et al. showed that in HPV16-positive CC, APC promoter 1A hypermethylation, rather than mutation, leads to β-catenin stabilization and activation of Wnt/β-catenin signaling. Demethylation restored APC expression and reduced oncogenic targets like MMP-7 and VEGF, implicating epigenetic silencing of APC in CC progression [144]. Chen et al. revealed that E6 promotes β-catenin transcriptional activity via the MZF1–NKX2-1–FOXM1 axis, where FOXM1 directly interacts with nuclear β-catenin to amplify oncogenic transcriptional output. These findings underscore a feed-forward regulatory loop, in which HPV oncoproteins not only initiate but also sustain β-catenin signaling, reinforcing malignant progression [145].

Clinical genomic analyses identified mutations or copy number variations in Catenin Beta 1 (CTNNB1), the gene encoding β-catenin, and alterations in other Wnt pathway regulators such as CREBBP, EP300, and TCF7L2 in 80% of cervical tumors, which further establishes the Wnt/β-catenin pathway as a frequently dysregulated signaling network in CC [143,146]. Chen et al. demonstrated that Wnt-11 is significantly upregulated in CC and is positively correlated with HR-HPV E6, advanced tumor stage, lymph node metastasis, and tumor size. Functional studies in HeLa and SiHa cells revealed that Wnt-11 promotes cell proliferation and invasion by activating the Wnt/JNK signaling pathway, as indicated by increased phosphorylation of JNK-1 (P-JNK1). Wnt-11 knockdown reduced P-JNK1 and β-catenin levels, while its overexpression enhanced these effects. These findings suggest that Wnt-11 facilitates CC progression through JNK1 activation and may serve as a potential therapeutic target [147]. HPV16/18 E6 oncoproteins promote CC progression by influencing both cancer cells and the tumor microenvironment through extracellular vesicles (EVs). E6 increases Wnt7b mRNA levels in CC cells and their EVs, which are taken up by Human Umbilical Vein Endothelial Cells (HUVECs), enhancing angiogenesis via β-catenin signaling [148].

Elevated serum EV-Wnt7b levels in patients are associated with aggressive disease and poor prognosis. As an independent prognostic marker, EV-Wnt7b supports a predictive nomogram for overall and recurrence-free survival, emphasizing its potential clinical value and the role of EVs in HPV-mediated tumor progression [149]. Collectively, these findings highlight Wnt/β-catenin as a downstream effector of HPV oncoproteins and also as a driver of oncogenic transcriptional programs in CC. Its activity influences tumor aggressiveness, stemness, resistance to therapy, and disease recurrence. Given its central role, therapeutic strategies targeting β-catenin signaling, such as small-molecule inhibitors, epigenetic modulators, or agents that disrupt β-catenin interactions, could be actively explored and represent a promising direction for treating HPV-associated cervical malignancies.

HPV oncoproteins E6 and E7 engage the canonical Wnt/β-catenin pathway to facilitate CC progression and metastasis. Uren et al. showed that activation of the Wnt/β-catenin pathway is essential for the malignant transformation of HPV-immortalized human keratinocytes. The authors of this study report that Wnt signaling, through β-catenin stabilization, cooperates with HPV to drive transformation, but is insufficient on its own. Invasive cervical cancer tissues showed strong β-catenin accumulation, unlike precancerous lesions, suggesting that Wnt activation contributes to cancer progression and may serve as a marker for metastasis [150]. E6 promotes β-catenin stabilization through several mechanisms: it blocks β-catenin ubiquitination via E6AP, disrupts the APC-mediated degradation complex by targeting PDZ-domain proteins, and activates the MZF1/NKX2-1/FOXM1 transcriptional axis.

This leads to nuclear accumulation of β-catenin and increased TCF-mediated expression of oncogenes such as c-Myc and Cyclin D1, and pluripotency markers like Nanog and Oct4, thereby promoting tumor progression. The Postsynaptic density protein 95 (PDZ)-binding motif of E6 plays a crucial role in activating Wnt signaling. K14-E6 transgenic mice have shown that this motif is necessary for increased expression of Wnt target genes and the development of invasive cervical lesions. In addition, E7 supports this process by inhibiting the phosphatase PP2A, which normally helps degrade β-catenin. By blocking PP2A, E7 promotes β-catenin stability and further amplifies Wnt-driven oncogenic signaling [151]. In a double-transgenic mouse model (K14-E7/ΔN87βcat), co-expression of HPV16 E7 with constitutively active β-catenin led to invasive CC in 94% of mice by seven months, which exceeded the tumor incidence seen with either activated β-catenin alone or E7 alone. Together, these findings highlight the interplay between HPV oncoproteins and the Wnt/β-catenin pathway in driving CC progression and metastasis [152]. Targeting this axis may offer a promising therapeutic approach for reversing disease advancement.

E6 and E7 influence immune evasion strategies that facilitate persistent infection and CC progression [153]. Aberrant activation of the Wnt/β-catenin pathway supports immune escape by stabilizing PD-L1 and suppressing T-cell infiltration. β-Catenin directly binds to the PD-L1 (CD274) and Suppressor of Tumorigenicity 3 (STT3) gene promoters, leading to increased expression of STT3 and enhanced N-glycosylation of PD-L1, which stabilizes PD-L1 protein and strengthens its immunosuppressive function [154]. β-catenin signaling helps tumors evade the immune system by promoting the survival of Tregs, which suppress the activity of CD8^+^ T cells. It also increases the expression of PD-L1 and blocks T-cell function, making the tumor more resistant to immunotherapy [155]. HPV E6 reduces the levels of MAGI3, a scaffold protein that controls Wnt/β-catenin signaling. When MAGI3 is suppressed, β-catenin builds up in the cell, leading to stronger Wnt signaling. This increase in Wnt activity contributes to the creation of an immunosuppressive tumor environment, helping CC cells evade immune responses [156].

E6 inhibits β-catenin degradation by interfering with the β-catenin destruction complex, particularly by downregulating seven in absentia homolog 1 (Siah-1), a p53-induced E3 ubiquitin ligase that targets β-catenin for degradation [157]. HPV E6 promotes the ubiquitin-mediated degradation of PDZ-domain tumor suppressors Dlg1 and Scribble, disrupting the APC/Axin/GSK3β complex integral to β-catenin turnover. Through its PDZ-binding motif and interaction with E6AP, E6 targets Scribble for degradation and induces Dlg1 degradation, attenuating the integrity of the β-catenin destruction complex. This leads to β-catenin stabilization and nuclear accumulation, facilitating aberrant activation of Wnt/β-catenin signaling in cancer cells [158]. E6 enhances transcription of the Wnt ligand WNT7B, increasing its mRNA levels in HPV-positive CC cells. This effect is mediated by EVs that shuttle WNT7B to endothelial cells, promoting angiogenesis through activation of β-catenin signaling. Knockdown of E6 reduces both cellular and extracellular WNT7B expression, confirming E6’s role in autocrine and paracrine activation of Wnt/β-catenin signaling in CC [159]. E7 binds to both the catalytic and structural subunits of PP2A, the serine/threonine phosphatase responsible for activating GSK3β within the β-catenin destruction complex. This binding inhibits PP2A activity, preventing GSK3β activation and leading to cytoplasmic stabilization of β-catenin. Consequently, β-catenin accumulates and activates Wnt signaling in HPV-positive CC cells [160].

For the downstream effector mechanism, β-catenin translocates to the nucleus where it binds to TCF/LEF transcription factors, activating tumor growth and survival. Among the key downstream targets are c-MYC, cyclin D1, survivin, and VEGF, which promote cell-cycle progression, inhibit apoptosis, and enhance angiogenesis [161]. FOXM1, a β-catenin/TCF-regulated transcription factor, is significantly upregulated in CC and enhances tumor cell proliferation, resistance to oxidative stress, and invasion. HPV-16/18 E6 induces FOXM1 expression via the MZF1/NKX2-1 axis, resulting in increased nuclear localization of β-catenin and activation of Wnt/β-catenin signaling. Elevated FOXM1 promotes the transcriptional activation of MMP-2/9, drives invasive behavior, and enhances stemness properties in vitro and tumor aggressiveness in vivo [162]. AXIN2, a well-known negative feedback regulator of Wnt signaling, is overexpressed in cervical neoplasia due to persistent β-catenin activation [163]. Immunohistochemical and mRNA analyses of CIN showed upregulation of AXIN2 alongside Wnt1, Wnt3a, and SNAIL, indicating active canonical Wnt pathway engagement during cervical carcinogenesis [164] (Figure 4D).

### 4.5. Cyclin-Dependent Kinases (CDKs) in the Pathogenesis of HPV-Induced Cervical Cancers

E6 and E7 of high-risk HPV are known to actively manipulate cyclin-dependent kinases (CDKs) to deregulate the cell-cycle and promote CC. E7, in particular, binds directly to CDK2–cyclin A/E complexes, independently of proteins like pRb, to accelerate G1/S transition, facilitating DNA replication and cell proliferation [165]. HPV E7 was shown to disrupt the G1 DNA damage checkpoint by promoting degradation of pRb, leading to uncontrolled E2F activity and S-phase entry. Studies also demonstrated that, despite the induction of Cdk inhibitors like p21, E7-expressing cells continued to proliferate, suggesting that E7 overrides inhibitory signals to promote genomic instability and cell cycle progression in CC [166]. Ajiro et al. investigated FIT-039, a selective CDK9 inhibitor, for its anti-HPV activity in cervical neoplasia. By inhibiting CDK9, a key regulator of transcription, FIT-039 suppressed HPV E6/E7 expression, restored p53 and pRb, and reduced viral replication. It effectively blocked HPV-induced dysplasia in CIN models and inhibited tumor growth in HPV-positive xenografts without notable toxicity, highlighting its potential as a therapeutic for HPV-driven cervical lesions [167].

HPV E7 was shown to disrupt the G1 DNA damage checkpoint by targeting and degrading pRB, leading to the release of E2F transcription factors and unscheduled entry into the S phase. Despite the induction of cell-cycle inhibitors such as p21 and p27, E7-expressing cells bypassed these regulatory blocks, promoting genomic instability and contributing to the malignant progression of CC [166]. CDK9 was found to be upregulated during CC and was positively associated with aggressive clinical features such as tumor size, stromal invasion, and lymph node metastasis. In HPV16-positive cancers, E6 contributed to CDK9 overexpression, which promoted cell proliferation by enhancing AKT2 signaling and suppressing p53 activity. The oncogenic role of CDK9 appeared to be mediated through the AKT2/p53 axis, suggesting its potential as a therapeutic target in CC [168]. Bi et al. demonstrated that MAGT1 (Magnesium Transporter 1) is crucial for the proliferation of HPV-positive CC cells. Knockdown of MAGT1 in HeLa and SiHa cells led to S-phase arrest and apoptosis, which is accompanied by decreased expression of CDK2 as well as cyclins D1 and E1, and reduced activation of the ERK/p38 MAPK pathway. The study also revealed that MAGT1 supports the function of HPV E6/E7 oncoproteins, highlighting its role in G1/S transition and oncogenic signaling [169]. These findings suggest that MAGT1 promotes CC progression by regulating cell-cycle machinery and MAPK-driven proliferation and may serve as a novel therapeutic target. Future research should focus on developing and optimizing selective CDK inhibitors, particularly targeting CDK2 and CDK9, for therapeutic use in HPV-driven CC. Investigating combination strategies involving CDK inhibitors with immune checkpoint blockers could enhance efficacy and overcome resistance.

CDK6 has been shown to play a critical role in metabolic adaptation and cell survival in CC. In CC cells, CDK6 activation enhances aerobic glycolysis and suppresses autophagy by phosphorylating the mTORC1–HK2 axis, thereby supporting tumor growth and metastatic behavior [170]. CDK9 is transcriptionally upregulated by E6 in CC and is positively correlated with advanced tumor stage and metastatic progression. Mechanistically, CDK9 facilitates transcriptional elongation and promotes cell survival by modulating the AKT2–p53 signaling axis. Functional inhibition of CDK9 has been demonstrated to significantly attenuate tumor proliferation and metastasis in vivo, underscoring its potential as a therapeutic target in HPV-driven cervical malignancies [171]. E7 upregulates regulator of chromosome condensation 1 (RCC1), a guanine nucleotide exchange factor, through E2F1-mediated transcriptional activation. RCC1, in turn, promotes CDK1 activation, facilitating cell-cycle progression through the G1/S transition, even in the presence of genotoxic stress, thereby enabling HPV-transformed cervical cancer cells to bypass DNA damage checkpoints and continue proliferating [172]. CDK1 plays a critical role in maintaining DNA replication and proper chromosome segregation during cell division. Meanwhile, the CDK5/cyclin I axis, activated in part by E6/E7-driven signaling, is linked to resistance against cisplatin treatment in CC, suggesting a role for CDK5 in both drug tolerance and the promotion of metastatic behavior [173].

E7 upregulates RCC1, which subsequently activates CDK1 through E2F1-dependent transcription [174]. This aberrant activation of CDK1 allows CC cells to override DNA-damage-induced checkpoints, promoting uncontrolled proliferation [175]. Critically, this checkpoint bypass is associated with impaired MHC class I-mediated antigen presentation, thereby facilitating immune evasion by reducing recognition and clearance by cytotoxic T lymphocytes [176]. While current evidence supports a link between E7-driven CDK1 activation and impaired immune surveillance in CC, further studies are needed to elucidate the precise molecular mechanisms by which CDK dysregulation affects antigen presentation and immune evasion, particularly within the HPV-infected tumor microenvironment.

E6 interacts with ubiquitin ligase E6AP, inducing proteasome-mediated degradation of p53, leading to loss of the G1/S and G2/M checkpoints and genomic instability [177]. Xu et al. revealed that E6 alters the expression of various cyclin-related proteins, including E2F5 and CDK5, both of which are upregulated, while G1S regulators are downregulated to promote uncontrolled cell-cycle entry [178]. E6 also interacts with tumor suppressor BRCA1-Associated RING Domain 1 (BARD1), which impacts DNA repair and checkpoint control independently of p53, leading to cancer progression [179]. E7 promotes dysregulation of cell-cycle progression through its high-affinity binding to pRb and related proteins, prompting their phosphorylation and degradation. This releases E2F, which in turn upregulates cyclins A and E, as well as downregulating CDK inhibitors p21^Cip1^ and p27^Kip1^, effectively removing inhibitory controls on CDK [180]. E7 influences other regulatory proteins such as histone deacetylases (HDACs), as well as transcription factors such as AP-1 and IRF-1, which further affect the expression of cyclins and CDK inhibitors [181]. E7 has been shown to induce upregulation of chaperones like HSP60, which are indirectly linked to cyclin-dependent signaling by promoting CC [182]. Gandhi et al. performed proteomic profiling to compare HPV16/18 E7-transfected keratinocytes with HPV-transformed CC cell lines and normal HaCaT cells. They observed enrichment of mTORC1 signaling, MYC targets, hypoxia, and glycolysis pathways. Phosphoglycerate kinase 1 (PGK1) emerged as a consistently upregulated protein across these pathways, highlighting its potential as a therapeutic target and an upstream regulator [183]. Yi et al., showed that E6 targets p53 for ubiquitin-mediated proteasomal degradation by reducing p21 expression and disrupting the G1/S checkpoint in CC [184].

In CC, oncogenic activation of the cyclin E–CDK2 complex drives a cascade of downstream events that promote cell proliferation, genomic instability, and tumor progression. A critical downstream substrate of CDK2 in CC is USP37, a deubiquitinase, and upon phosphorylation at Ser628, it enhances the stability of ERK1/2 kinases. This modification sustains activation of the RAF–MEK–ERK signaling cascade, thereby amplifying mitogenic signaling and promoting tumor cell proliferation and growth in CC [185]. Downstream targets of CDK2 in CC include p53, Cdc25A/C, MDM2, BRCA1, and E2F1, whose phosphorylation contributes to checkpoint bypass, impaired DNA repair, and enhanced proliferation [186]. Downstream effector p21^Cip1^, controlled by the enzyme Lysine (K)-specific Demethylase 6A (KDM6A), helps protect HPV E7-expressing CC cells from replication stress. HPV E7 protein increases KDM6A levels, which then turn on the p21^Cip1^ gene. Subsequently, p21^Cip1^ slows down DNA replication by blocking Proliferating Cell Nuclear Antigen (PCNA). When KDM6A or p21^Cip1^ is removed, the cells undergo uncontrolled DNA replication, severe DNA damage, and apoptosis [187]. Collectively, E6 and E7 orchestrate multilayered disruption of cell-cycle regulation in CC by targeting both upstream tumor suppressors and downstream effectors of cyclin-dependent kinase pathways (Figure 4E).

## 5. Molecular Mechanisms of Kinase Regulation by HPV Oncoproteins

E6 and E7 intricately modulate host kinase signaling networks to facilitate malignant transformation and progression of CC. These viral proteins engage both direct and indirect mechanisms to activate or inhibit critical kinase cascades such as the PI3K/Akt/mTOR, MAPK/ERK, and JNK pathways, thereby rewiring cellular processes that govern proliferation, survival, and immune evasion. E7 oncoprotein of HPV-16 reportedly activates the PI3K/Akt signaling pathway in CC cells. E7 enhanced the phosphorylation of Akt (PKB) independently of PTEN inhibition or PI3K activation [188]. E7 is bound to PP2A subunits, which prevent Akt dephosphorylation and sustain its activation. This mechanism supports virus-driven cell survival and contributes to the development of cancer [189].

Lee et al. explored how the HPV16 E6 oncoprotein promotes CC progression by upregulating the Axl receptor tyrosine kinase. The authors of this study demonstrated that E6 enhanced the expression of Axl by modulating the PTEN/AKT pathway and inducing MZF1 transcriptional activity. Axl, in turn, facilitates metastasis and immune evasion. Targeting Axl signaling is proposed as a promising therapeutic strategy against HPV-associated CC [190]. Another study by Zhou et al. found that E6 of HPV16 targets and degrades the tumor suppressor TSC2, leading to increased phosphorylation of S6 kinase, independent of Akt activation, indicating an alternative mechanism of mTOR pathway activation in HPV-positive tumors [191]. Collectively, these findings highlight the multifaceted role of HPV oncogenes in hijacking kinase signaling cascades to promote cervical carcinogenesis. Strickland et al. found that E7 of HPV-16 suppresses AKT phosphorylation in keratinocytes independently of Rb degradation, altering downstream targets like S6K and 4E-BP1 and enhancing IRES-dependent translation in CC. The study highlighted stage-specific modulation of the PI3K/AKT pathway during HPV-induced carcinogenesis [192]. Garcia et al. demonstrated how HPV E6 oncoproteins modulate eIF4E activity and showed that E6 from various HPV genotypes, particularly high-risk ones, increased phosphorylation of eIF4E at Ser209 via activation of the PI3K/AKT and ERK pathways. This upregulation enhanced protein synthesis of oncogenic targets like CCND1 and ODC1, suggesting that E6 promotes cervical carcinogenesis by modifying translational control mechanisms [120]. In SCC and HSIL, the EGFR/PI3K/Akt/mTOR pathway is known to be significantly activated. Increased EGFR expression leads to activation of mTOR and its downstream effector p70S6K. This signaling cascade promoted nuclear translocation of phosphorylated mTOR and p70S6K, which correlated with increased Ki-67, Skp2, and mitotic activity expression. These findings suggest that EGFR-mTOR signaling contributes to CC by driving uncontrolled cell-cycle progression, and may serve as a target for therapeutic intervention [193].

E6 and E7 also directly engage kinase signaling pathways, such as by activating the MEK/ERK/MNK1/eIF4E axis, leading to oncogenic protein translation and resistance to growth-inhibitory signals [96]. Simultaneously, E6/E7 oncoproteins promote JNK and c-Jun phosphorylation, driving EMT and reinforcing EGFR feedback loops, a process that is reversible with JNK pathway inhibition [194]. Under hypoxic conditions, PI3K/mTORC2-mediated Akt signaling maintains suppression of E6/E7, ensuring cancer cell dormancy and resistance, emphasizing the contextual plasticity of kinase activity in HPV-driven tumors [195]. Figure 3 illustrates HPV E6-mediated degradation of p53, and E7-induced inhibition of pRb, which disrupts cell-cycle checkpoints, enabling sustained proliferation and evasion of apoptosis. 

In summary, these mechanistic insights illustrate a dual strategy employed by HPV, disabling tumor suppressors to release inhibitory barriers while activating oncogenic kinases to sustain proliferation, survival, immune evasion, and metabolic shifts. Such multifaceted regulation underscores key vulnerabilities in the PI3K/Akt/mTOR, MEK/ERK, and JNK pathways, presenting multiple actionable targets for precision therapy in HPV-associated CC.

## 6. Mechanisms of Drug Resistance in HPV-Driven Cervical Cancers

HPV oncoproteins E6 and E7 orchestrate multiple mechanisms of therapeutic resistance in CC. It has been reported that E6-mediated degradation of p53 via E6AP disables the key DNA damage response pathway and impairs cisplatin-induced apoptosis [196]. HPV oncoproteins promote immune evasion through PD-L1 upregulation. Studies have shown the functional role of HPV16 E6-driven pathways, such as miR-143/HIF-1α and NF-κB/PI3K/Akt, in the elevation of PD-L1 expression on tumor cells, fostering an immunosuppressive microenvironment that further impairs chemotherapy responses [105]. E6 and E7 activate the cGAS–TOP1 (topoisomerase I) axis, stabilizing TOP1 and activating cGAS-driven NF-κB signaling, which enhances PD-L1 expression and disrupts DNA repair mechanisms. Inhibition of TOP1 in CC cells impairs repair and reduces tumor growth. Konstantopoulos et al. explored the role of HPV16 E6 in immune evasion in CC. E6 was knocked out using CRISPR/Cas9 in HPV16-positive cell lines, and reduced the expression of the immune checkpoint protein PD-L1 and its transcription factor HIF-1α, alongside increased levels of miR-143, a known HIF-1α suppressor. Analyses of these findings suggest that E6 promotes immune escape by regulating PD-L1 via the miR-143/HIF-1α axis [197]. It is known that persistent HPV16 infection plays a critical role in CC progression, largely due to the immunosuppressive effects of its viral oncoproteins E6 and EThese proteins not only disrupt tumor suppressor pathways but also contribute to immune escape mechanisms that allow CC cells to evade immune surveillance. Lin et al. identified a key regulatory axis involving microRNA miR-142-5p and the immune checkpoint molecule PD-LUsing bioinformatic screening followed by functional validation, miR-142-5p was shown to be significantly downregulated upon E6/E7 expression in CC cells, while PD-L1 was upregulated. Gain- and loss-of-function experiments confirmed that miR-142-5p directly targets PD-L1, thereby acting as a tumor suppressor by inhibiting its expression [105]. Furthermore, upregulation of miR-142-5p was able to reverse E6/E7-induced PD-L1 overexpression, thereby reducing immune evasion. In vivo studies demonstrated that restoring miR-142-5p expression suppressed tumor growth and prolonged survival in mouse models, highlighting the functional significance of the miR-142-5p/PD-L1 axis [198].

Therapeutic strategies aimed at restoring miR-142-5p levels may enhance anti-tumor immunity and serve as a novel immunotherapeutic approach in CC. Putral et al. investigated whether RNA interference (RNAi) targeting HPV oncogenes E6 and E7 could sensitize CC cells to chemotherapy. Small interfering RNAs (SiRNAs) against E6 simultaneously suppressed E7 expression, resulting in an 80% reduction in E7 protein and reactivation of the tumor suppressor pThis dual inhibition reduced cell viability and induced senescence in HPV-positive HeLa cells, with no impact on HPV-negative cells. Importantly, the combination of shRNA targeting E6 with cisplatin significantly enhanced chemosensitivity. The combinatorial treatment also led to greater p53 accumulation and E7 suppression than either treatment alone, suggesting that silencing viral oncogenes can potentiate the efficacy of conventional chemotherapy through restoration of tumor suppressor pathways [199]. Reddy et al. recently developed an in vitro model of chemo-radioresistant CC using HPV16-positive SiHa cells to replicate clinical treatment conditions involving cisplatin and ionizing radiation. The resulting resistant cells displayed significantly higher resistance and IC50 values, along with elevated expression of HPV16 E6/E7 oncoproteins. The study evaluated the repurposed drug Nelfinavir, an HIV protease inhibitor, and found that it effectively sensitized resistant cells. Mechanistically, Nelfinavir suppressed the AKT pathway and reduced levels of USP15, USP11, and HPV16 E6/E7 proteins. Knockdown of USP15 and USP11 by siRNAs further confirmed their role in maintaining E6/E7 expression [200]. These findings suggest that Nelfinavir targets the AKT–USP15/USP11–E6/E7 axis, offering a promising strategy to overcome therapy resistance in CC.

## 7. Therapeutic Strategies Targeting HPV and HPV-Induced Kinase Pathways in Cervical Cancer

Current therapeutic approaches for CC increasingly aim to target both E6 and E7 and the dysregulated kinase signaling pathways modulated by these proteins, which include CDKs, MAPKs, and the PI3K/Akt/mTOR axis, to inhibit tumor growth, restore tumor-suppressive mechanisms, and overcome treatment resistance [201]. Therapeutic targeting of E6 and E7 using CRISPR/Cas9 was effective in HPV16- and HPV18-transformed CC cells, as this approach resulted in the gene-specific cleavage of E6 or E7, thereby decreasing the oncogenic properties while promoting tumor-suppressive mechanisms. The resulting mutations disrupted viral oncogene expression and restored p53 or Rb tumor suppressor activity, which has led to cell-cycle arrest and death [202]. This study provided proof-of-concept that Cas9/sgRNA combinations delivered via viral vectors could selectively eliminate HPV-positive cancer cells by inactivating the integrated viral genome, offering a novel gene-based therapeutic strategy [203].

Park et al. demonstrated that HIV-1 aspartyl protease inhibitors, such as Lopinavir, Nitonavir, Nelfinavir, and Saquinavir, can selectively reduce HPV16 E6 and E7 oncoprotein levels in HPV16-transformed CaSki cells and organotypic raft cultures [204]. This downregulation increased p53 expression, reduced viability of HPV16-positive cells, and reversed HPV-induced autophagy disruption, without affecting HPV-negative models. Among the tested inhibitors, Saquinavir showed the most pronounced and selective long-term effects. These findings suggest that repurposing of HIV-1 protease inhibitors could be a novel therapeutic strategy for HPV16-associated CC [205]. The combined effect of the HSP70 inhibitor SHetA2 and the CDK4/6 inhibitor Palbociclib on HR-HPV-positive CC cells was determined, focusing on modulation of E6/E7 oncoproteins and associated signaling pathways. The combination therapy led to marked downregulation of HPV E6/E7 expression and upregulation of host AP-1 factors (c-Jun and c-Fos), surpassing the effects of either drug alone [206]. This was accompanied by decreased AKT/mTOR phosphorylation, indicating disruption of HPV-driven survival signaling. These findings suggest a promising therapeutic strategy targeting HPV oncogenesis via combinatorial pathway inhibition for further preclinical and clinical evaluation [207]. Table 2 lists the therapeutic interventions against HPV-associated kinase pathways in CC.

Nichols et al. identified and optimized small-molecule inhibitors such as E6AP that covalently bind to a specific cysteine within the HPV-16 E6 oncoprotein. These compounds disrupted E6–E6AP interaction, restored p53 levels, and selectively reduced viability in HPV-16-positive CC cells without affecting HPV-negative cells. In vivo, administration of these compounds was achieved by injecting SiHa, UM-SC-47, and UM-SCC-104 cancer-derived cells subcutaneously into the flanks of immunodeficient mice. This achieved >70% tumor growth inhibition in HPV-16 xenograft models. This study highlighted a novel, targeted therapeutic strategy against HPV-16-driven CC that works by directly inactivating E6 [212]. A combination of E6-specific siRNA with the chemotherapeutic agent Oxaliplatin targeting HPV16-positive CC cells enhanced p53-dependent apoptosis, reduced stemness and metastasis, and significantly sensitized cells to Oxaliplatin, leading to apoptosis and cell-cycle arrest in the sub-G1 phase. This combination therapy allowed for lower drug dosage while maintaining efficacy, supporting its potential as an effective and targeted CC treatment approach [213]. Silencing of the HPV16 E6 oncogene by siRNA restored p53 function and enhanced the sensitivity of HPV-positive CC cells to various apoptosis-inducing treatments. E6 suppression increased p53 and p21 expression, sensitizing cells specifically to cisplatin-induced apoptosis, while having a limited effect on apoptosis induced by irradiation, rhTRAIL, or anti-Fas antibody alone. However, combining Cisplatin with rhTRAIL or anti-Fas in E6-silenced cells significantly enhanced apoptosis via caspase activation and XIAP downregulation. These findings highlighted a potential therapeutic strategy involving E6 or XIAP inhibition to potentiate Cisplatin- and death receptor-mediated apoptosis in HPV-positive CC [214]. Xie et al. demonstrated that Punicalagin, a compound from pomegranate peel, suppressed CC by promoting autophagy-dependent degradation of HPV oncoproteins E6 and EThis took place through the activation of the ROS–JNK pathway, which disrupts the BCL2–BECN1 complex and induces autophagy [215]. Inhibiting autophagy or JNK, or scavenging ROS, prevented E6/E7 degradation. Punicalagin effectively reduced tumor growth in vivo, highlighting its therapeutic potential in HPV-driven CC [216].

Emerging evidence highlights the potential of plant-derived compounds (phytopharmaceuticals) in targeting kinases that are activated by HPV oncoproteins, particularly E6- and E7-driven signaling pathways, which promote CC. Phytochemicals capable of modulating these kinase pathways offer promising strategies for CC prevention and therapy. Zhao et al. investigated the effects of Curcumin on HPV-positive CC cells (HeLa and CaSki) and found that Curcumin significantly inhibited cell viability, migration, and invasion in a dose- and time-dependent manner. It induced apoptosis via the mitochondrial pathway and modulated key apoptotic and EMT markers. Curcumin downregulated E6 and E7, especially E6, highlighting its potential as a therapeutic agent [217]. These findings suggest that Curcumin’s anticancer effects are in part mediated through E6 suppression and mitochondrial apoptotic signaling.

Glycyrrhizin is another natural compound isolated from licorice root [218]. It showed strong antiproliferative and pro-apoptotic activity in HPV16-positive CaSki cells. Mechanistically, Glycyrrhizin was able to induce ROS generation, disrupt mitochondrial function, and activate caspase-8 and -9, which led to the induction of apoptosis. It also caused G0/G1 cell-cycle arrest by modulating cyclin D1/CDK4 and p21 expression. Glycyrrhizin also downregulated E6 and E7 oncoproteins and suppressed the Notch signaling pathway, highlighting its potential as a targeted therapeutic agent against HPV-driven CC [219]. Einbond et al. evaluated the anti-HPV effects of various plant-derived compounds on W12 cervical precancer cells, which carry HPV16 DNA. Among the tested agents, Tanshinone IIA and Curcumin were found to be the most potent ones, significantly inhibiting the expression of HPV16 oncogenes E1, E2, E4, E6, and E7, and restoring p53 expression. The combination of Curcumin and Tanshinone IIA demonstrated synergistic induction of apoptosis in HeLa cells. Molecular docking indicated that both compounds might be acting through the binding to the Na^+^/K^+^-ATPase ion channel, suggesting a novel bioelectric signaling mechanism underlying their activity [220].

The above-mentioned studies highlighted the therapeutic promise of phytochemical-based combinatorial approaches in preventing and treating HPV-induced CC. Supporting these studies, a recent investigation evaluating the effect of Epigallocatechin-3-gallate (EGCG), a major green tea polyphenol, has reported growth-inhibitory properties when tested against CaSki cells. EGCG inhibited cell proliferation in a dose-dependent manner and induced significant apoptosis. Mechanistically, EGCG exerted its apoptosis-inducing effects by downregulating 16 genes while upregulating 4 genes, indicating its role in modulating gene expression [221]. Supporting these in vitro studies, experiments carried out using in vivo models also showed anti-tumor properties of EGCG, positioning it as a promising phytopharmaceutical agent for treating HPV-related CC.

Sun et al. investigated the anticancer effects of Resveratrol on CC, focusing on its ability to suppress viral oncogene expression. The authors of this study demonstrated that Resveratrol could inhibit the expression of E6 mRNA and protein, reduce pRb1, and restore the p53 protein levels in both HeLa and CaSki cells, as well as in tumors derived from HeLa xenografts. Resveratrol disrupted the bicistronic RNA-mediated expression of E6 and E7 oncogenes, resulting in cell-cycle arrest at the G1/S transition and induction of apoptosis [222]. These findings highlighted the potential of Resveratrol as a therapeutic agent targeting HPV-driven CC. Furthermore, they also investigated the anticancer effects of Resveratrol on HPV-positive CC and showed that it had a viral oncogene-suppressive effect. These findings highlight Resveratrol’s potential as a therapeutic agent targeting HPV-driven cervical carcinogenesis. Collectively, these studies illustrate how phytopharmaceuticals target E6 and E7 proteins, suppressing oncogenic signaling and reactivating tumor-suppressor networks.

Targeting HPV-driven kinase signaling and HPV-targeted gene editing or proteolysis strategies (PROTAC and CRISPR/Cas) offers a promising multimodal approach for CC therapy. Khairkhah et al. investigated the therapeutic potential of CRISPR/Cas9 gene editing in targeting E6 and E7 in HPV16-related CC. CRISPR/Cas9 constructs were designed targeting E5, E6, E7, and the p97 promoter and delivered into C3, TC1, and HeLa cells [223]. The authors observed that CRISPR-mediated editing, particularly the combined targeting of E6 and E7, led to significant tumor growth suppression, apoptosis induction, and cell-cycle arrest via restoration of p53, Rb, and p21 expression. In vivo experiments further confirmed the antitumor efficacy, with the E6 + E7 targeting group showing the most pronounced reduction in tumor size and high levels of cleaved caspase-3, indicating effective apoptosis. Zhen et al. explored the synergistic potential of combining CRISPR/Cas9-mediated targeting of HPV16 E6/E7 with PD-1 immune checkpoint blockade in CC. CRISPR/Cas9 targeting of HPV16 E6/E7 in SiHa cells induced apoptosis and reduced PD-L1 expression, which was elevated earlier by E6/EIn humanized SCID mice, co-administration of gRNA-HPV16 E6/E7 with gRNA-PD-1 significantly suppressed tumor growth, improved survival, and increased CD8^+^ and CD4^+^ T cells, as well as dendritic cell populations. The combination also reprogrammed the tumor microenvironment by upregulating Th1-stimulatory genes and downregulating immunosuppressive pathways, demonstrating robust immune-mediated antitumor activity [224].

Li et al. designed a CRISPR/Cas9 nanoeditor that targeted and deleted the E6 and E7 oncogenes to overcome chemotherapy resistance in CC. By targeting these oncogenes, they reactivated p53 and pRB tumor suppressor pathways, leading to inhibited tumor growth. The nanoeditor was combined with Docetaxel using cationic liposomes, resulting in significantly improved treatment effects. The study showed that this dual-target gene editing approach could be a powerful strategy for treating drug-resistant CC [225].

Recent advances in targeted protein degradation have led to the development of PROTACs (proteolysis-targeting chimeras) as a promising class of therapeutics for HPV-driven CC [226]. Smalley et al. engineered a nanobody-based PROTAC, named PROTAC^E6^, to selectively degrade the HPV16 E6 oncoprotein in CC models. This degrader effectively reduced tumor growth in immunocompetent mice, restored innate immune signaling, and triggered apoptosis in CaSki cells due to E6 degradation via the VHL E3 ligase pathway [227]. A novel stapled peptide-based PROTAC (SP-PROTAC) was designed to target the palmitoyltransferase ZDHHC3, thereby promoting the degradation of PD-L1 in CC cells. SP-PROTAC significantly reduced PD-L1 levels in HeLa and C33A cell lines, outperforming the known PD-L1 inhibitor BMS-Mechanistically, the effect was shown to be proteasome-dependent, as it was reversed by the inhibitor MGAdditionally, in co-culture with T cells, SP-PROTAC enhanced immune activation by increasing IFN-γ and TNF-α production [228]. Future directions should focus on translating these targeted approaches into clinical trials. Emphasis on E6/E7 gene editing, siRNA delivery, kinase pathway inhibition, CRISPR/Cas, and PROTAC technologies alone and in combination with immunomodulators or checkpoint inhibitors must be evaluated clinically to target CC (Table 3).

## 8. Kinase Pathways and Drug Resistance in Cervical Cancer

Although advances in screening and the widespread implementation of HPV vaccination have improved early detection and prevention, therapeutic resistance, especially to platinum-based chemotherapy and radiotherapy, continues to limit clinical outcomes. The aberrant regulation of kinase signaling pathways that govern essential cellular processes, such as survival, proliferation, and the DNA damage response, leads to drug resistance. Elucidating how these kinase pathways are altered in CC is therefore crucial for identifying vulnerabilities and guiding the development of more effective targeted therapies.

### 8.1. PI3K/AKT/mTOR Pathway, Aurora Kinases, and Chemoresistance in Cervical Cancer

In CC, E6 stabilizes ARKA, leading to its overexpression and dysregulated activity, which contributes to chromosomal instability and tumor progression [80]. This aberrant activation facilitates bypass of DNA-damage-induced cell-cycle arrest, rendering tumor cells less susceptible to apoptosis triggered by chemotherapeutic agents such as cisplatin [91]. Pharmacologic inhibition of ARKA has been shown to induce G2/M arrest and promote apoptosis, thereby sensitizing CC cells to cisplatin treatment [88]. Additionally, Aurora kinase inhibition attenuates NF-κB signaling, which is implicated in promoting survival and drug resistance mechanisms [215].

The PI3K/AKT/mTOR pathway regulates fundamental cellular processes, including metabolism, growth, and survival. Activating mutations and amplifications in the PIK3CA gene, which encodes the catalytic subunit of PI3K, are common in CC, especially in advanced and metastatic tumors [107]. The hyperactivation of this pathway enables an anti-apoptotic cellular milieu, enhances DNA repair capacity, and drives metabolic reprogramming that supports cell survival under cytotoxic stress [237]. Targeted inhibition of PI3K/AKT/mTOR signaling restores chemosensitivity in preclinical CC models, and combination therapies involving pathway inhibitors and conventional chemotherapeutics have demonstrated synergistic tumor growth suppression [238]. Though Aurora kinases and PI3K/AKT/mTOR constitute separate signaling cascades, their activities converge in promoting CC cell survival and drug resistance [239]. All this evidence suggests potential crosstalk between Aurora kinases and PI3K/AKT signaling components, underlining the possibility that combined therapeutic targeting may yield enhanced anticancer efficacy against CC.

### 8.2. MAPK Pathway, Aurora Kinases, and Drug Resistance in Cervical Cancer

The mitogen-activated protein kinase (MAPK) signaling pathway, encompassing ERK, JNK, and p38 kinases, is central to the control of cellular proliferation, differentiation, stress responses, and apoptosis [240]. Aberrant activation of MAPK cascade components is frequently observed in CC and has been closely linked to tumor progression and resistance to chemotherapy and radiotherapy [241]. Phosphorylation-induced activation of ERK enhances DNA repair mechanisms and cell survival following cytotoxic insult, thereby reducing apoptosis and promoting chemoresistance [242].

In CC cells, elevated MAPK/ERK signaling confers drug resistance through modulation of anti-apoptotic proteins such as Bcl-2 and the upregulation of multidrug efflux transporters, including P-glycoprotein, which diminishes intracellular drug accumulation. Pharmacological inhibition of MAPKs sensitizes cancer cells to cisplatin and radiation therapy, disrupting the pro-survival signaling required for resistance [138]. Aurora kinases, primarily Aurora A, also contribute significantly to CC chemoresistance through mechanisms that complement MAPK pathway functions. Aurora A facilitates tumor cell proliferation, chromosomal instability, and evasion of apoptosis, often by stabilizing oncogenic proteins and promoting mitotic progression, even under DNA-damaging conditions induced by chemotherapy [243]. The interplay between Aurora kinases and MAPK signaling components may influence the phosphorylation state and activity of ERK and other MAPK family members, amplifying pro-survival signals that could also act on CC [244]. This synergy could enhance resistance phenotypes and tumor aggressiveness.

Combined pharmacological inhibition of both MAPK signaling and Aurora kinase activity could thus represent a potent therapeutic approach, disrupting multiple axes of tumor cell survival and overcoming resistance mechanisms in CC [245]. Such multi-targeted strategies may improve patient outcomes by preventing the compensatory pathway activation that typically undermines single-agent therapies.

### 8.3. Role of p53 and Kinase Crosstalk in Drug Resistance in Cervical Cancer

The tumor suppressor p53 is an important regulator of cellular responses to genotoxic stress, including DNA-damage-induced apoptosis, cell-cycle arrest, and senescence [246]. In CC, p53 function is commonly compromised by the HPV oncoprotein E6, which recruits the E3 ubiquitin ligase E6AP to induce proteasomal degradation of p53, effectively abrogating its tumor-suppressive activities [247]. This loss disrupts critical DNA damage checkpoints, enabling CC cells to survive chemotherapy and radiotherapy, which induce DNA lesions.

Aurora A intersects significantly with p53 signaling. Aurora A directly phosphorylates p53 on Ser215 and Ser315 residues, which inhibits p53’s transcriptional activity and enhances MDM2-mediated p53 degradation [248]. This kinase-driven modulation further diminishes p53’s ability to induce apoptosis and cell-cycle arrest in response to chemotherapy [249]. Aurora kinases also phosphorylate hnRNPK, a transcriptional co-activator of p53, reducing its function and thus impairing the p53-mediated genotoxic stress response [250].

Mutant or functionally inactivated p53 not only fails to activate pro-apoptotic pathways but may gain oncogenic functions that promote drug resistance by upregulating multidrug resistance genes like MDR1 [251]. Restoring p53 activity, either via gene therapy approaches using recombinant adenovirus-p53 (rAd-p53) or small molecules that inhibit p53 degradation, has been found to resensitize CC cells to chemotherapeutics. The combination of rAd-p53 with lobaplatin was found to enhance apoptosis and tumor growth inhibition in CC cells [252].

The cross-regulation between p53 and kinases extends to pathways such as PI3K/AKT/mTOR and MAPK, which also influence p53 stability and function. mTOR inhibition has been shown to induce p53-dependent apoptosis, increasing chemotherapy sensitivity [253]. Conversely, constitutive kinase signaling can suppress p53 activity, driving therapy resistance [254].

The combination of therapeutic strategies that restore or activate p53 and inhibit key oncogenic kinases such as Aurora A can lead to enhanced apoptosis and tumor regression [251]. Thus, targeting the kinase-p53 axis offers a promising avenue to overcome drug resistance in HPV-driven cervical cancers.

## 9. Clinical Trials Targeting HPV-Induced Kinase Pathways in Cervical Cancer

Recent clinical efforts have focused on targeting kinase-driven signaling pathways that are aberrantly activated in HPV-associated CC. These targeted therapies aim to disrupt critical nodes contributing to tumor growth, chemoresistance, and metastatic spread. Only a limited number of early-phase clinical trials have investigated dosage regimens and combinations with existing treatment options. Table 4 lists the ongoing clinical trials targeting HPV-induced kinase pathways in CC.

## 10. Conclusions and Future Perspectives

This review highlights the interplay between HR-HPV oncoproteins, E6 and E7, and host kinase signaling pathways that collectively drive CC. These viral proteins not only disrupt tumor suppressor networks involving p53 and Rb but also activate several kinases such as PI3K/Akt/mTOR, MAPK/ERK, Wnt/β-catenin, and CDKs, thereby facilitating proliferation, immune evasion, metabolic rewiring, and drug resistance. The oncogenic persistence of HPV is further reinforced through feedback loops, epigenetic modifications, and evasion of apoptosis, contributing to therapy failure and tumor progression. Despite advances in molecular understanding and targeted therapies, resistance to chemotherapy and limited clinical translation of kinase-targeted interventions remain significant challenges. Future research must focus on understanding key mechanistic and translational gaps. Comprehensive multi-omics studies, including proteomics and spatial transcriptomics, are needed to unravel the dynamic regulation of HPV-activated kinases across disease stages and genotypes. Special emphasis should be placed on identifying temporal patterns and co-activation signatures that could pave the way to biomarker-driven therapy. Dissecting the interactions between HPV-infected cells and the tumor microenvironment is essential. Understanding how kinases mediate stromal remodeling, immune suppression, and angiogenesis will enable the development of combination therapies that target both tumor-intrinsic and -extrinsic pathways. Co-culture systems, 3D organoids, and patient-derived xenografts will be important tools in this aspect.

The development of precise, less toxic therapeutic agents targeting E6/E7 or their downstream effectors should be explored. Gene editing tools and siRNA-based approaches also require optimization of delivery systems, including nanoparticles and viral vectors, for HPV-tropic specificity and clinical feasibility. In conclusion, tackling HPV-driven CC requires a multifaceted approach involving deep molecular characterization, advanced computational tools, and innovative therapeutics. By addressing these domains, it will ultimately contribute to the WHO’s vision of CC elimination.

## Figures and Tables

**Table 1:** List of available HPV vaccines and their cost in India.

S. No.	Vaccines	Manufacturer	Targeted HPV Genotypes	Cost (Rs)	Reference
1	Cervavac	Serum Institute of India (Pune, India)	HPV 6,11,16,18	300–400	[33]
2	Gardasil 4	Merck & Co. (Rahway, NJ, USA)	HPV 6, 11, 16, 18	3000–4000	[34]
3	Gardasil 9	Merck & Co. (Rahway, NJ, USA)	HPV 6, 11, 16, 18, 31, 33, 45, 52, 58	10,000–11,000	[35]

**Table 2:** Chemoresistance mechanisms and their molecular modulators in CC.

Mechanism	Details	Intervention/Effect	Reference
Overexpression of drug efflux pumps (MRP1, P-gp)	Cisplatin-resistant SiHaR cells show high MRP1 and P-gp levels, reducing intracellular drug accumulation by 20–70%	Curcumin inhibits HDAC1/2, downregulates MRP1/P-gp, and restores cisplatin sensitivity	[208]
Enhanced DNA repair via NER and PARP-1	Increased ERCC1 and metallothioneins, and PARP-1 expression leads to cisplatin resistance	Targeting DNA repair pathways may reverse resistance	[209]
Anti-apoptotic Bcl-2 family overexpression	HeLa cisplatin-resistant cells show elevated Bcl-2, Bcl-xL, Bag-1, and Mcl-1, diminishing drug-induced apoptosis	Bcl-2/Bcl-xL silencing restores Beclin-1, activates caspase-3/7, and resensitizes cells to cisplatin	[210]
Cyclin I–Cdk5 axis activation	Cisplatin-resistant cervical tumors overexpress cyclin I, which activates Cdk5, preventing apoptosis	Cdk5 inhibition or cyclin I knockdown restores cisplatin sensitivity, increasing apoptosis both in vitro and in vivo	[173]
Autophagy-mediated resistance (ATG5/ATG7)	Autophagy protects cervical cancer cells from cisplatin or paclitaxel by clearing cytotoxic stress and promoting survival	ATG5 or ATG7 silencing (siRNA) or 3-MA/chloroquine treatment enhances apoptosis and restores drug sensitivity	[211]

**Table 3:** Targeted therapeutic interventions against HPV-associated kinase pathways in cervical cancer.

Strategy	Target	Pharmacological Agent(s)	Key Outcomes	Reference
CDK9 inhibition	CDK9—transcriptional regulator of E6/E7	FIT-039	↓ E6/E7, ↑ p53/pRb, suppressed CIN dysplasia, and inhibited tumor growth with minimal toxicity	[167]
CDK2/CDK1 inhibition	CDK2/1—cell-cycle kinases subverted by E7	Roscovitine, Purvalanol	Induced G1 arrest and apoptosis in HPV-positive cell lines and restored cell-cycle control	[229]
JNK inhibition	JNK1/2—activated by E6	SP600125 (JNK inhibitor)	↓ c-Jun and EGFR/ERK signaling, and reduced proliferation, EMT, and E6/E7 expression in cervical cancer models	[77]
Autophagy activator via JNK	JNK-mediated autophagy degradation of E6/E7	Punicalagin	Promoted JNK–BCL2-mediated autophagic degradation of E6/E7 and suppressed tumor growth in vivo	[230]
Dual PI3K/mTOR inhibition	PI3K/mTOR—key survival pathway	BEZ235, BKM120	Overcame rapamycin resistance, induced apoptosis in HPV-positive models, and enhanced cisplatin efficacy	[231]
CDK4/6 inhibition	CDK4/6–Rb–E2F axis	Abemaciclib	Was effective in HPV-negative cervical cancer via cell-cycle arrest, though less so in HPV-positive settings	[232]
mTORC1 combination therapy	mTORC1 + AMPK	Everolimus + Metformin	Synergistic inhibition of proliferation, ↑ apoptosis, and ↑ p38/ERK/JNK signaling	[233]
p38 MAPK inhibition	p38 MAPK-mediated apoptosis	DEPTOR	Induced apoptosis	[234]
Pentose phosphate pathway (PPP) inhibition	G6PD (via E6-mediated PPP activation)	6-Aminonicotinamide (6-An)	E6 activated PPP by inhibiting G6PD lactylation; 6-An blocked proliferation and tumor growth	[235]
AKT–TKT(Transketolase) metabolic axis	TKT (via AKT activation by E6)	Oxythiamine + cisplatin	E6-induced AKT activation boosted TKT in PPP; OT reduced tumor growth and synergized with cisplatin	[236]

**Table 4:** Key Clinical Trials Targeting Kinase Pathways in Cervical Cancer.

Clinical Trial Number	Intervention	Target Pathway	Patient Population	Key Outcome	Reference
NCIC CTG IND 199	Temsirolimus (CCI-779)	AKT/mTOR	Recurrent/metastatic cervical cancer (Phase II)	Modest activity in cervical carcinoma	[255,256]
NCT07038369	ATV-1601	AKT	Phase 1, open-label study	Not yet determined	
NCT01226316	AZD5363	AKT1/PIK3CA or PTEN mutation	Phase 1, open-label study	Not yet determined	-

## Data Availability

No new data were created or analyzed in this study.

## Abbreviations

The following abbreviations are used in this manuscript:

ARKC	Aurora kinase C
4EBP1	4E Binding Protein 1
ACLY	ATP-citrate lyase
AMPK	5′ adenosine monophosphate-activated protein kinase
AP	Activator Protein-1
APC	Adenomatous Polyposis Coli
ARKA	Aurora kinase A
ARKB	Aurora kinase B
ASK1–p38 MAPK	Apoptosis Signal-Regulating Kinase 1- p38 Mitogen-Activated Protein Kinase
ATM	Ataxia-Telangiectasia Mutated
BAD	BCL2-Associated agonist of cell death
Bax,	Bcl-2-associated X protein
BCL-2	B-cell lymphoma 2
BRCA1	BReast CAncer gene 1
c-Fos	Finkel–Biskis–Jinkins Osteosarcoma
c-Jun	Cellular Jun Oncogene
CC	Cervical cancer
CCND1	Cyclin D1
CDK1	Cyclin-dependent kinase 1
cGAS	Cyclic GMP-AMP Synthase
CIN	Cervical intraepithelial neoplasia
cMET	Mesenchymal–Epithelial Transition factor
CREBBP	cAMP Response Element Binding Protein
CTNNB1	Catenin Beta 1
CXCR4	Chemokine receptor type 4
Dlg	Discs Large
DNA	Deoxyribonucleic acid
DUSP5	Dual-Specificity Phosphatase 5
E	Early region
E6AP	E6-associated protein
EGF	Epidermal growth factor
EGFR	Epidermal growth factor receptor
ELK1	ETS-Like 1
EMT	Epithelial–Mesenchymal Transition
EP300	E1A Binding Protein P300
ER-α36	Estrogen receptor- α36
ERK	Extracellular Signal-Regulated Kinase
FAK	Focal adhesion kinase
FHIT	Fragile Histidine Triad
FOXM1	Forkhead box protein M1
FOXO	Forkhead box O
GDP	Guanosine diphosphate
GPCRs	G protein-coupled receptors
GRB2	Growth factor receptor-bound protein 2
GSK3a/b	Glycogen synthase kinase3-a/b
GSK3β	Glycogen Synthase Kinase 3 beta
GTP	Guanosine triphosphate
HBD2	Human Beta-Defensin 2
HCIL	High-grade cervical intraepithelial neoplastic lesions
HER2	Human Epidermal growth factor Receptor 2
HICs	High-income countries
HIF-1α	Hypoxia-inducible factor 1-alpha
HIV	Human immunodeficiency virus
HMGA2	High Mobility Group AT-hook 2
HPV	Human Papilloma Virus
HR -HPV	High risk Human Papilloma Virus
Hsp90	Heat Shock protein 90
HSV	Herpes simplex virus
HUVECs	Human Umbilical Vein Endothelial Cells
IGF-1R	Insulin-like growth factor 1 receptor
IGF1	Insulin-like Growth Factor 1
IL-8	Interleukin 8
JNK	c-Jun N-terminal kinase
LR	Late region
LCR	Long control region
LEF	Lymphoid enhancer factor
LMICs	Low- and middle-income countries
LRP1B	Low-density lipoprotein receptor-related protein 1B
MAGT1	Magnesium Transporter 1
MAPK	Mitogen-activated protein kinase
MCL	Myeloid Cell Leukemia
MHC	Major histone complex
miR	Micro RNA
MMP-9	Matrix metalloproteinase-9
MNK	MAPK-Interacting Kinase
mTOR	Mammalian target of Rapamycin
mTORC2	Mammalian target of rapamycin complex 2
MYC	Myelocytomatosis viral oncogene
MYC	Myelocytomatosis oncogene
MZF1	Myeloid Zinc Finger 1
MZF1–NKX2-1–FOXM1	Myeloid Zinc Finger 1–NK2 Homeobox 1–Forkhead Box M1
NF-κB	Nuclear Factor kappa B
NK	Natural killer
NR4A2	Nuclear Receptor Subfamily 4 Group A Member 2
nu	Nude
ODC1	Ornithine Decarboxylase 1
ori	Origin of replication
P-JNK1	Phosphorylation of JNK-1
PDGF	Platelet-derived growth factor
PDGFR	Platelet-derived growth factor receptor
PDL 1	Programmed death-ligand 1
PDZ	The Postsynaptic density protein 95
PI3K	Phosphotidylinositol–trisphosphate kinase
PIK3CA	Phosphatidylinositol-4,5-bisphosphate 3-kinase catalytic subunit alpha
PIP2	Phosphatidylinositol 4,5-bisphosphate
PIP3	Phosphatidylinositol 3,4,5-trisphosphate
PKC	Protein Kinase C
PLD	Phospholipase D
PP2A	Protein Phosphatase 2A
pRb	Retinoblastoma protein
PTEN	Phosphatase and TENsin
Puma	p53 upregulated modulator of apoptosis
Rb	Retinoblastoma
RCC1	Regulator of Chromosome Condensation 1
RTKs	Receptor tyrosine kinases
S6K	S6 kinase
Ser	Serine
SH2	Src homology
Siah-1	Seven in absentia homolog 1
SOS	Son of sevenless
Sp1	Specificity protein 1
STARD3	StAR-related lipid transfer domain-containing 3
STING	Stimulator of Interferon Genes
STT3	Suppressor of Tumorigenicity 3
TCF	T-cell factor
TCF7L2	Transcription factor 7 like 2
Thr	Threonine
TIGAR	TP53-inducible glycolysis and apoptosis regulator
TNF-α	Tumor necrosis factor-alpha
URR	Upstream regulatory region
VEGF	Vascular endothelial growth factor
WHO	World Health Organization
Wnt	Wingless-related integration site
FIT	N-[5-fluoro-2-(1-piperidinyl) phenyl] isonicotinthioamide
E2F	Early 2 factor transcription factor
Hk2	Hexokinase 2
BARD1	BRCA1 Associated RING Domain 1
HDACs	Histone deacetylases
PGK1	Phosphoglycerate kinase 1
E–CDK2	Cyclin E/Cyclin-dependent kinase 2
USP37	Ubiquitin-specific processing protease 37
MDM2	Murine Double Minute 2
Cdc25A/C	Cell Division Cycle 25A/C
KDM6A	Lysine (K)-specific Demethylase 6A
PCNA	Proliferating Cell Nuclear Antigen
PKB	Phosphorylation of Akt
IRES	Internal Ribosome Entry Site
Skp2	S-phase kinase-associated protein 2
TOP1	Topoisomerase I
RNAi	RNA interference
SiRNAs	Small interfering RNAs
USP	Ubiquitin-Specific protease
MRP1	Multidrug Resistance Protein 1
P-gp	P-glycoprotein
PARP-1	Poly(ADP-ribose) polymerase-1
NER	Nucleotide excision repair
Bag-1	BCL2-associated athanogene 1
ATG	Autophagy protein
sgRNA	Single guide RNA
rhTRAIL	Recombinant human tumor necrosis factor (TNF)-related apoptosis-inducing ligand
ROS	Reactive oxygen Species
ATP	Adenosine triphosphate
EGCG	Epigallocatechin-3-gallate
PROTAC	Proteolysis-targeting chimera
SCID	Severe combined immunodeficiency
CRISPR	Clustered Regularly Interspaced Short Palindromic Repeats
gRNA	Guide RNA
SP-PROTAC	Stapled peptide-based proteolysis-targeting chimera
